# Extracellular vesicles derived from creeping fat stem cells promote lymphatic function and restrain inflammation of Crohn's disease

**DOI:** 10.1002/ctm2.70086

**Published:** 2024-12-02

**Authors:** Weigang Shu, Yongheng Wang, Mengfan Chen, Xiaoli Zhu, Fangtao Wang, Chunqiu Chen, Peng Du, Alexandra Bartolomucci, Xin Su, Xiaolei Wang

**Affiliations:** ^1^ Department of Gastroenterology Shanghai Tenth People's Hospital School of Medicine Tongji University Shanghai China; ^2^ Department of General Surgery Shanghai Tenth People's Hospital School of Medicine Tongji University Shanghai China; ^3^ Department of Colorectal Surgery Xinhua Hospital, Shanghai Jiaotong University School of Medicine Shanghai China; ^4^ Cancer Research Program Research Institute of McGill University Health Center Montreal Canada

**Keywords:** adipose‐derived stem cells, creeping fat, Crohn's disease, extracellular vesicle, lymphatic function

## Abstract

**Background:**

Crohn's disease (CD) is a chronic inflammatory disease in the intestinal tract. Mesenteric fat wrapping and thickening, or creeping fat (CrF), is a typical characteristic of CD and it involves lymphangiogenesis and altered lymphatic function. By releasing extracellular vesicles (EVs), adipose tissue‐derived stem cells (ADSCs) can regulate their adjacent cells. However, the regulating roles of ADSC‐EVs in CrF (CrF‐EVs) in CD, especially in modulating lymphatic function and mitigating the progression of mesenteritis and colitis, remains elusive.

**Methods:**

To evaluate the regulative roles of CrF‐EVs on lymphatic functions, in vitro assays were performed using human lymphatic endothelial cells (HLECs). Next, Interleukin 10 knock‐out (*Il‐10* ^−/−^) mice were used to assess the biological functions of CrF‐EVs in spontaneous mesenteritis and colitis. Moreover, tissue and serum from various cohorts of CD patients were used to determine the prognostic value of miR‐132‐3p.

**Results:**

CrF‐EVs significantly attenuated spontaneous mesenteritis and colitis in *Il‐10* ^−/−^ mice via promoting lymphangiogenesis and lymphatic drainage. Using high‐throughput sequencing, we demonstrated that CrF‐EVs significantly increased HLEC proliferation, migration, tube formation and CCL‐21 production in a miR‐132‐3p/RASA1/ERK1/2 axis‐dependent manner. Accordingly, upregulated miR‐132‐3p was observed in patient CrF, positively correlated with lymphangiogenesis while negatively correlated with inflammatory factors (tumour necrosis factor‐α and IL‐6) level. Moreover, serum miR‐132‐3p demonstrated a positive correlation with disease activity.

**Conclusions:**

EVs derived from CrF ADSCs, containing elevated levels of miR‐132‐3p, could promote lymphatic function and restrain inflammation of CD. Our results provide a novel insight into the role of mesenteric lymphatics in CD progression and reveal a new potential therapeutic.

**Key points:**

- Extracellular vesicles (EVs) of creeping fat (CrF) derived adipose stem cells effectively attenuate chronic mesenteritis and colitis in Crohn's disease (CD).
- The lymphatic vessels play an important role in disease development of CD and their functions are improved by CrF‐EV‐miR‐132‐3p through RASA1/ERK1/2 signaling.
- MiR‐132‐3p expression is upregulated in CrF and serum of CD patients, and tightly linked with inflammation and disease activity.

## Graphical abstract

1. Extracellular vesicles (EVs) derived from adipose tissue‐derived stem cells in creeping fat (CrF) improve lymphatic functions and increase mesenteric lymphatic vessel density of *Il‐10* ^‐/–^ mice, leading to decreased colitis and mesenteritis through the miR‐132‐3p/RASA1 /ERK1/2 axis.

2. Co‐culturing human lymphatic endothelial cells with CrF‐EVs promotes proliferation, migration and tube function in vitro.

3. Upregulated miR‐132‐3p in CrF was negatively correlated with proinflammatory cytokines. Moreover, serum miR‐132‐3p was positively correlated with disease activity.

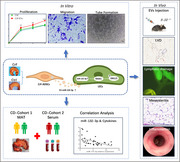

## 1 INTRODUCTION

The CD is a chronic, relapsing gastrointestinal disease characterized by inflammation throughout the intestinal wall and surrounding structures (such as the mesentery). Creeping fat (CrF) is the wrapping of hypertrophic mesenteric adipose tissue (MAT) around segments of the inflamed gut and is an important yet poorly understood hallmark of CD.^1^, ^2^ However, recent studies suggest that CrF may play a protective role in CD development.^3^, ^4^, ^5^

The inflamed and lipid‐rich microenvironment in CrF can lead to abnormal function and appearance of cells.^6^ Namely, adipocytes in CrF often expand in large numbers and are usually typified by a smaller size and increased expression of both pro‐ and anti‐inflammatory cytokines and adipokines.^7^ Within the cellular components, adipose‐derived stem cells (ADSCs) are important stromal cells that exhibit potent regulatory properties and pro‐inflammatory roles.^8^ In addition, recent studies showed that ADSCs from CrF (ADSC‐CrF) could display a potential transformation of the browning phenotype, which is demonstrated as a compensatory change in the regulation of intestinal inflammation in CD patients.^5^, ^9^ Moreover, ADSCs can communicate with surrounding cells through extracellular vesicles (EVs), including microvesicles, EVs and apoptotic bodies.^10^, ^11^ In fact, a previous study has demonstrated that CrF‐ADSCs activated fibroblasts through EV delivery.^12^

Lymphatic vessels (LVs) transport lymph, proteins, immune cells and digested fats into the lymph nodes. The abnormal morphology and function of the mesenteric components in CD could alter lymphatic drainage and adipokines/cytokines production, leading to intestinal inflammation and disease progression.^13^ Oedema, lymphangiogenesis, immune cell trafficking impairments and lymph leakage can all result from impaired lymph drainage. MAT lymph leakage intensifies inflammation by stimulating adipose tissue growth and the production of cytokines, which affect immune cell functions. This is regarded as one of the critical mechanisms of CrF formation.^14^

The CD is a chronic disease with obvious dysfunctions in the lymphatic vessel system.^13^ Malfunction of lymphatic drainage may contribute to the impaired integrity and high permeability of lymphatic endothelial cells (LECs), resulting in the aggregation of inflammatory mediators and the proliferation of adipocytes.^15^, ^16^ In addition, lymphangiogenesis and increased lymphatic vessel density (LVD) induced by vascular endothelial growth factor (VEGF)‐C/VEGF receptor‐3 (VEGFR‐3) signalling have been observed in the inflammatory intestine and CrF.^17^, ^18^ LECs are the main source of CCL21, which promotes the alleviation of inflammation through the migration of immune cells out of inflamed tissues by binding to its receptor CCR7.^19^, ^20^ Recent studies have shown that the resection of CrF affects the postoperative recurrence of CD.^17^, ^21^ Moreover, increased LVD at the resection margin and improved lymphatic functions could effectively attenuate intestinal inflammation.^22^, ^23^ Drug delivery targeting mesenteric lymphatic vessels also improved lymphatic drainage and decreased intestinal inflammation.^24^ Additionally, EVs can be uptaken by the lymphatic system and can significantly regulate lymph node functions in pathological conditions.^25^, ^26^, ^27^ Therefore, we hypothesized that EVs secreted by CrF‐derived ADSCs could regulate mesenteric lymphatic functions, and eventually affect mesenteritis and colitis.

In this study, CrF‐EV‐miR‐132‐3p promoted lymphangiogenesis and lymphatic drainage function via the RASA1/ERK1/2 axis, which effectively attenuated colitis and mesenteritis in CD. Moreover, our study revealed that miR‐132‐3p levels were higher in CD patients than in healthy controls and inextricably linked with the Crohn's Disease Activity Index (CDAI). This research not only provides novel insights into the role of CrF in CD progression but also highlights the potential value of miR‐132‐3p as a diagnostic biomarker of CD. Importantly, the results of this study pave the way for future work to focus on the bioengineering of vectors loaded with miR‐132‐3p targeting lymphatic vessels, as this holds significant therapeutic potential both in the treatment of mesenteritis and intestine inflammation of CD.

## 2 METHODS

### 2.1 Ethical considerations

Ethics approval was obtained from Shanghai Tenth People's Hospital's Institutional Ethics Committee (SHSY‐IEC‐4.1/20‐152/01 and SHDSYY‐2019‐3886). For the animal study, Shanghai SLAC Laboratory Animal Co., Ltd provided us with female C57BL/6 wild‐type (WT) mice (15 weeks old) with a body weight of 25–30 g. Female *Il‐10* ^−/−^ mice were generated by a commercial supplier (Cyagen Biosciences, Santa Clara, CA, USA). The mice were kept in a second‐class animal facility at Tongji University's Laboratory Animal Center in Shanghai, China, following a standard 12‐h light/dark cycle. Guidelines for the Care and Use of Laboratory Animals were followed during animal experiments, and sterile food and water were provided. Informed consent was obtained from all participants in accordance with approved guidelines for the study.

### 2.2 Patients and samples

The MAT, intestine and serum samples were collected between January 2020 and December 2022 from the Shanghai 10th People's Hospital and the Shanghai Jiaotong University Xinhua Hospital. CrF and the corresponding inflammatory intestine samples were obtained from 25 patients (cohort 1) who received resection of intestine segments and the paired non‐CD MAT. The normal MAT and the intestine tissues as control (Ctrl) were acquired surgically from 10 colon cancer patients. For the serum analysis, samples were collected from another 30 CD patients (cohort 2) and 10 healthy controls (HC). The CD activity index (CDAI) was used to assess disease activity and dichotomize patients into active (CDAI > 150) and remission (CDAI 0–150) groups. Exclusion criteria included those who were under 18 years old, unwilling to provide informed consent, or undergoing chemoradiation. Clinical and demographic data of the patient cohort are detailed in Supplementary Materials.

### 2.3 Statistics analysis

For accuracy and replicability, all experiments were conducted in triplicate. Continuous variables are presented as means ± standard deviation (SD) unless specified otherwise. A one‐way analysis of variance or unpaired Student's t‐test was used to determine statistical significance. We used SPSS and Prism software for the statistical analysis. A correlation analysis using Spearman's correlation was conducted. A *p*‐value of  .05 was considered statistically significant.

## 3 RESULTS

### 3.1 EVs from ADSC‐CrF circulate into the mesentery and colon of *Il‐10* ^−/−^ mice

**FIGURE 1:**
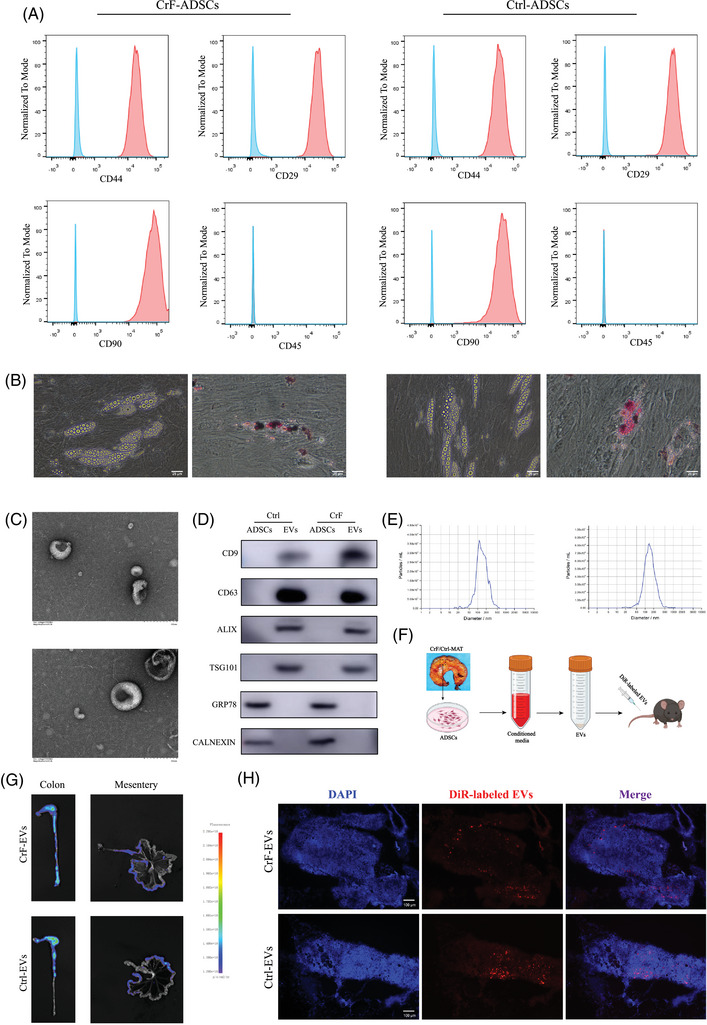
Extracellular vesicles (EVs) from mesenteric adipose tissue‐adipose tissue‐derived stem cells (MAT‐ADSCs) circulate into the mesentery and colon of *Il‐10* ^−/−^ mice. (A) Flow cytometry characterization of ADSCs from MAT. (B) Adipose differentiation of ADSCs from MAT by Oil Red O staining. (C) Transmission electron microscopy exhibited a typical structure of EVs. (D) EV protein expressions of TSG101, CD63, CD9, ALIX, GRP78 and CALNEXIN detected by Western blot. (E) Nanoparticle tracking analysis revealed the size of EVs. (F) The EVs labelled with DiR dye were injected into *Il‐10* ^−/–^ mice by tail vein. (G) DiR‐labelled EVs were detected in the intestine and mesentery by an imaging system. (H) IF staining validated the DiR‐labelled EVs in the mesentery of *Il‐10* ^−/–^ mice.

ADSCs were isolated from the CrF and normal MAT (Ctrl‐MAT) of 25 CD patients and cultured as described previously.^18^ The classical mesenchymal positive markers CD90, CD44, CD29 and the negative marker CD45 were confirmed in ADSCs by flow cytometry (Figure 1A). To further confirm the differentiation potential of ADSCs, both lipid droplet and Oil Red O staining were detected in differentiated CrF ADSCs and Ctrl‐MAT ADSCs (Figure 1B). The CrF‐EVs and Ctrl‐EVs demonstrated typical EV morphology, as observed by transmission electron microscopy (TEM) (Figure 1C). The expression of the common EV markers CD9, CD63, TSG101 and Alix^28^ were confirmed by western blot, while the expression of the GRP78 and CALNEXIN were exclusively detected in CrF‐ADSCs and Ctrl‐ADSCs rather than their EVs (Figure 1D), which demonstrated the high purity of the obtained EVs. Nanoparticle tracking analysis (NTA) demonstrated that the sizes of both CrF‐EVs and Ctrl‐EVs ranged from 100–200 nm (Figure 1E).

*Il‐10^−/−^* mice are a widely recognized animal model for spontaneous colitis and mesenteritis, mimicking human CD.^29^ To investigate the influence of CrF‐EVs on CD progression, the DiR fluorescence labelled EVs were injected into *Il‐10^−/−^* mice through the tail vein (Figure 1F). After 12 h, the fluorescence signal of DiR was detected in the mesentery and colon of *Il‐10^−/−^* mice using fluorescence microscopy and the uptaken signal displayed no differences between CrF‐EVs and Ctrl‐EVs (Figure 1G and Figure S1). Furthermore, IF staining confirmed considerable DiR‐labeled EVs in the mesenteric tissue. Thus, both CrF‐EVs and Ctrl‐EVs effectively localized in the targeted colon and mesentery of *Il‐10^−/−^* mice. (Figure 1H).

### 3.2 CrF‐EVs attenuate spontaneous colitis and mesenteric inflammation in *Il‐10* ^−/−^ mice

**FIGURE 2:**
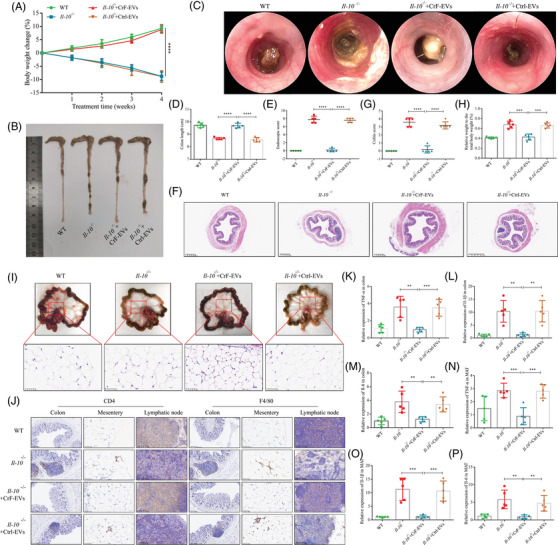
Creeping fat‐extracellular vesicles (CrF‐EVs) attenuate spontaneous colitis and mesenteric inflammation of *Il‐10* ^−/−^ mice. (A) The weight curve of mice during the four‐week injection of extracellular vesicles (EVs). (B) The representative photos of the colon. (C) The endoscopic findings. (D) The colonic length analysis. (E) Endoscopic score of colitis. (F) HE staining of the colon. (G) Histological score of colitis. (H) The ratio of mesenteric weight to body weight. (I) The representative photos of the mesentery. (J) IHC staining of F4/80^+^ macrophages and CD4^+^ T cells in colon, mesentery and lymph node. (K–M) Relative mRNA expression of Tnf‐α (K), Il‐1β (L) and Il‐6 (M) in the colon. (N–P) Relative mRNA expression of Tnf‐α (N), Il‐1β (O) and Il‐6 (P) expression in mesentery. Data are expressed as means ± SD. *p < .05, **p < .01, ***p < .001, ****p < .0001 and *n* = 5 mice in each group.

We observed that *Il‐10* ^−/−^ mice displayed overt inflammation in the colon and mesentery starting from 15 weeks old, which is in line with previous reports.^23^, ^24^ At week 19, *Il‐10^−/−^* mice exhibited spontaneous weight loss and shortened colonic length compared to WT mice (Figure 2A,B,D). In addition, using a high‐resolution endoscopic system, we observed that *Il‐10^−/−^* mice exhibit thickened colon walls and vascular pattern changes (Figure 2C,E). These phenotypes in *Il‐10^−/−^* mice correlated with higher endoscopic and histological scores compared to those in WT mice (Figure 2F,G). However, these changes could be alleviated through the administration of CrF‐EVs but not Ctrl‐EVs for 4 weeks.

The mesentery of *Il‐10^−/−^* mice also presented significant hypertrophy as reported previously.^29^ The significantly higher ratio of MAT weight to body weight was detected in *Il‐10^−/−^* mice in comparison to WT mice, which could be reversed by CrF‐EV treatment (Figure 2H,I). Interestingly, we found the MAT of *Il‐10^−/−^* mice exhibited adipocytes of smaller size than WT mice after CrF‐EV injection (Figure 2G and Figure S2A). This is a feature of adipose browning as an anti‐inflammatory mechanism in the MAT of CD which has recently been reported.^30^ Thus, we then sought to investigate the expression of uncoupling protein‐1 (Ucp‐1), a classic marker of adipose browning in mice. We found increased expression of Ucp‐1 protein in the MAT of *Il‐10^−/−^* mice receiving CrF‐EVs compared to Ctrl‐EVs (Figure S2B,C). Our findings suggested that there was a tendency for adipose browning after CrF‐EV treatment, indicating a potential protective role of CrF in inflammation.

Macrophages and CD4^+^ T cells are the two most common cellular infiltrates in CrF.^29^ Using immunohistochemistry (IHC) staining, we found an increased number of CD4^+^ T cells and F4/80^+^ macrophages in both the colon and the mesentery of *Il‐10^−/−^* mice compared to WT mice (Figure 2J and Figure S3A–D). CrF‐EVs rather than Ctrl‐EVs effectively abrogated this increased infiltration and significantly increased the numbers of CD4^+^ T cells and F4/80^+^ macrophages in the mesenteric lymph nodes of *Il‐10^−/−^* mice (Figure 2J and Figure S3E,F). These results indicate that CrF‐EVs may facilitate the emigration of immune cells from inflamed sites to lymph nodes, which attenuates inflammation. In addition, *Il‐10* ^−/−^ mice had elevated levels of proinflammatory factors, such as TNF‐α, Il‐1β and Il‐6 in the mesentery and intestine, and these pathological changes can be reverted after 4 weeks of CrF‐EVs administration (Figure 2K–P). In line with that, CrF‐EVs injection could also revert the elevated levels of serum TNF‐α and Il‐6 in *Il‐10^−/−^* mice (Figure S4). Collectively, our data exemplifies that CrF‐EVs treatment effectively attenuates disease progression and spontaneous inflammation in the mesentery and colon of *Il‐10^−/−^* mice.

### 3.3 CrF‐EVs enhance lymphatic drainage and LEC functions

**FIGURE 3:**
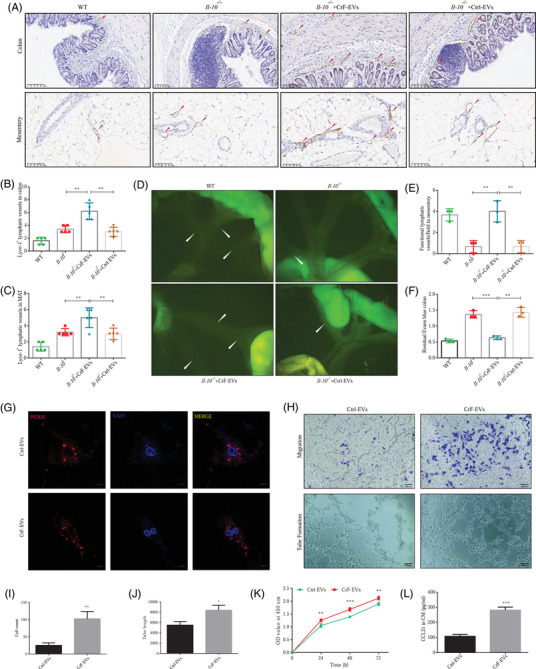
Creeping fat‐extracellular vesicles (CrF‐EVs) enhance lymphatic endothelial functions in vivo and in vitro. (A) Lyve‐1 positive lymphatic endothelium in the colon and mesentery in mice by IHC staining. (B, C) Lymphatic vessel density (LVD) analysis in the colon (B) and mesentery (C). (D) Functional lymphatic vessels were detected after oral administration of BODITY dye. (E) Quantification of functional lymphatic vessels in mesentery. (F) Lymphatic drainage in the colon detected by Evans blue dye. (G) EVs uptaken by human lymphatic endothelial cells (HLECs) in vitro. (H) HLECs migration and tube formation with CrF‐EVs incubation. (I, J) Quantification of HLECs migration (I) and tube formation (J) with CrF‐EVs incubation. (K) HLECs proliferation with CrF‐EVs incubation detected by Cell Counting Kit‐8 (CCK‐8). (L) ELISA analyzed CCL21 secretion by HLECs incubated with CrF‐EVs. Data are expressed as means ± SD. *p < .05, **p < .01, ***p < .001 and *n* = 5 mice in each group.

Since previous studies have shown that *Il‐10^−/−^* mice exhibited obvious lymphatic vessel dysfunction,^23^, ^24^ and our previous studies also confirmed that enhanced lymphatic drainage function could relieve colonic inflammation in a sodium trinitrobenzene sulfonate (TNBS)‐induced chronic colitis model,^18^ we hypothesized that improvement of mesenteric lymphatic function could effectively mitigate intestinal and mesenteric inflammation. ADSCs have been proven to improve LEC function by EV delivery in vitro.^26^ Herein, we further investigated whether CrF‐EVs affect colitis and mesenteritis in *Il‐10^−/−^* mice through the regulation of lymphatic functions. Lyve‐1, a specific marker of LECs, was used to assess lymphatic vessel density. After CrF‐EV treatment, the LVD of both the colon and mesentery in *Il‐10^−/−^* mice were significantly increased, exemplifying an influence on lymphangiogenesis (Figure 3A–C).

Seven hours after intragastric administration of Bodipy FL‐C16, a fluorescent lipid tracer, *Il‐10^−/−^* mice demonstrated impaired mesenteric lymphatic drainage compared with WT mice, as evidenced by the weaker fluorescence signal in the mesenteric lymphatic vessels of WT mice (Figure 3D). CrF‐EV‐treated *Il‐10^−/–^* mice demonstrated a distinguished increase in mesenteric lymphatic drainage compared with Ctrl‐EV treatment (Figure 3E). Moreover, we evaluated the extraction ability of Evans blue, a dye that is selectively absorbed by the intestinal lymphatic vasculature, among the different groups. Sixteen hours following submucosal injection into the distal rectum, the extraction of Evans blue dye was markedly impaired in *Il‐10^−/–^* mice compared to WT mice. Notably, these findings were reversible by systemic delivery of CrF‐EVs, as less dye remained in the distal rectum of CrF‐EV treated *Il‐10^−/−^* mice (Figure 3F).

To further validate the regulation of CrF‐EVs on lymphatic endothelium functions, the EVs were labelled with the fluorescence dye PKH26 and co‐incubated with HLECs for 24 h in vitro. Under confocal fluorescence microscopy, we observed that both CrF‐EVs and Ctrl‐EVs were uptaken by HLECs (Figure 3G). Moreover, HLECs co‐incubated with CrF‐EVs had significantly enhanced proliferation, migration and tube formation functions (Figure 3H–K). In addition, we also found that HLECs had remarkably increased CCL21 production after CrF‐EVs injection (Figure 3L). Interestingly, we also collected ADSC‐EVs from subcutaneous adipose tissue (SAT‐EVs) and stimulated HLECs. As expected, SAT‐EVs displayed no significant effects on lymphatic function or on CCL21 expression in LECs compared with Ctrl‐EVs (Figure S5). This data highlights the potent and exclusive regulatory roles of CrF‐EVs on LEC functions and immune cell migration. Taken together, our results demonstrate that CrF‐EVs can enhance lymphatic physiology, outgrowth and immunoregulatory functions both in vivo and in vitro.

### 3.4 CrF‐EVs improve lymphatic endothelial functions through miR‐132‐3p

**FIGURE 4:**
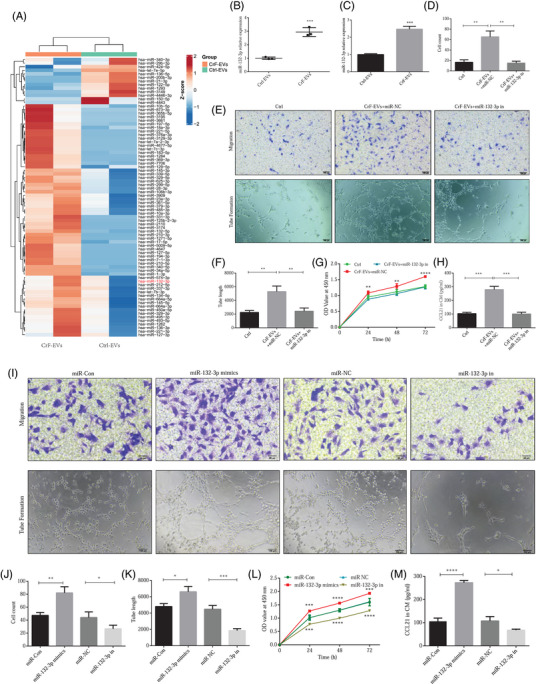
Creeping fat‐extracellular vesicles (CrF‐EVs) improved lymphatic endothelial functions through miR‐132‐3p delivery. (A) Heatmap of microRNAs (miRNAs) high throughput sequencing of CrF‐EVs and Ctrl‐EVs. (B) Quantitative polymerase chain reaction (qPCR) detected relative miR‐132‐3p expression in CrF‐EVs and Ctrl‐EVs. (C) miR‐132‐3p expression in human lymphatic endothelial cells (HLECs) with CrF‐EVs incubation. (D) Quantification of HLECs migration with CrF‐EVs‐miR‐132‐p inhibition. (E) HLECs migration and tube formation with CrF‐EVs‐miR‐132‐p inhibition. (F) Quantification of HLECs tube formation with CrF‐EVs‐miR‐132‐p inhibition. (G) Cell Counting Kit‐8 (CCK‐8) detected HLECs proliferation with CrF‐EVs‐miR‐132‐p inhibition. (H) CCL21 secretion by HLECs with CrF‐EVs‐miR‐132‐p inhibition. (I) HLECs migration and tube formation with miR‐132‐3p mimics or inhibitor transfection. (J, K) Analysis of HLECs migration (J) and tube formation (K) with different miR‐132‐3p expression. (L) HLECs proliferation with different miR‐132‐3p expression. (M) CCL21 secretion in a conditional medium of HLECs with miR‐132‐3p mimics or inhibitors. Data are expressed as means ± SD. *p < .05, **p < .01 and ***p < .001.

MicroRNAs (miRNAs) have been reported to be the most common mediator for executing EV functions.^31^, ^32^, ^33^ In order to clarify the molecular mechanisms underlying the regulation of CrF‐EVs in the lymphatic system, we performed miRNA high‐throughout sequencing on CrF‐EVs and Ctrl‐EVs. The heatmap obtained exhibited 47 upregulated miRNAs and 17 downregulated miRNAs in CrF‐EVs compared to Ctrl‐EVs [fold‐change ≥ 1.5, *p*  <  .05] (Figure 4A). Although the top 3 miRNAs (miR‐10b‐5p, miR‐873‐3p and miR‐365b‐5p), with the most significantly increased expression levels in CrF‐EVs, had been validated by quantitative polymerase chain reaction (qPCR), they exhibited no effects on lymphatic functions (Figure S6). Interestingly, miR‐132‐3p, which was reported in EVs derived from ADSCs in our previous study,^26^ was also highly expressed in CrF‐EVs compared to Ctrl‐EVs. We thus investigated its role as a regulator of LEC function. Indeed, we further confirmed its elevated expression in both CrF‐derived‐ADSCs and ADSC‐derived‐EVs (Figure 4B and Figure S7A). Furthermore, HLECs incubated with CrF‐EVs also displayed the upregulated level of miR‐132‐3p (Figure 4C). Thus, we inferred that CrF‐EVs regulate lymphatic functions via the delivery of miR‐132‐3p.

To further validate our hypothesis, we transfected miR‐132‐3p inhibitors into CrF‐EVs and co‐incubated them with HLECs. HLEC proliferation, migration and tube formation induced by CrF‐EVs were markedly suppressed by miR‐132‐3p inhibition (Figure 4D–G). Moreover, CCL21 secretion induced by CrF‐EVs in HLECs was also reversed after inhibition of EVs’ miR‐132‐3p (Figure 4H).

Next, we investigated whether miR‐132‐3p in HLECs directly affects cellular functions. MiR‐132‐3p mimics and inhibitors were transfected into HLECs, respectively. As expected, miR‐132‐3p levels were significantly upregulated in HLECs transfected with the miR‐132‐3p mimics (Figure S7B). The functions of HLECs including proliferation, migration and tube formation were also significantly enhanced accordingly. In contrast, these cellular functions and the secretion of CCL21 were suppressed after inhibition of miR‐132‐3p (Figure 4I–M). Thus, our results substantiated that CrF‐EVs improve lymphatic functions by miR‐132‐3p delivery.

### 3.5 CrF‐EV‐miR‐132‐3p promotes lymphatic endothelial functions by targeting RASA1 and activating ERK1/2 signalling pathway

**FIGURE 5:**
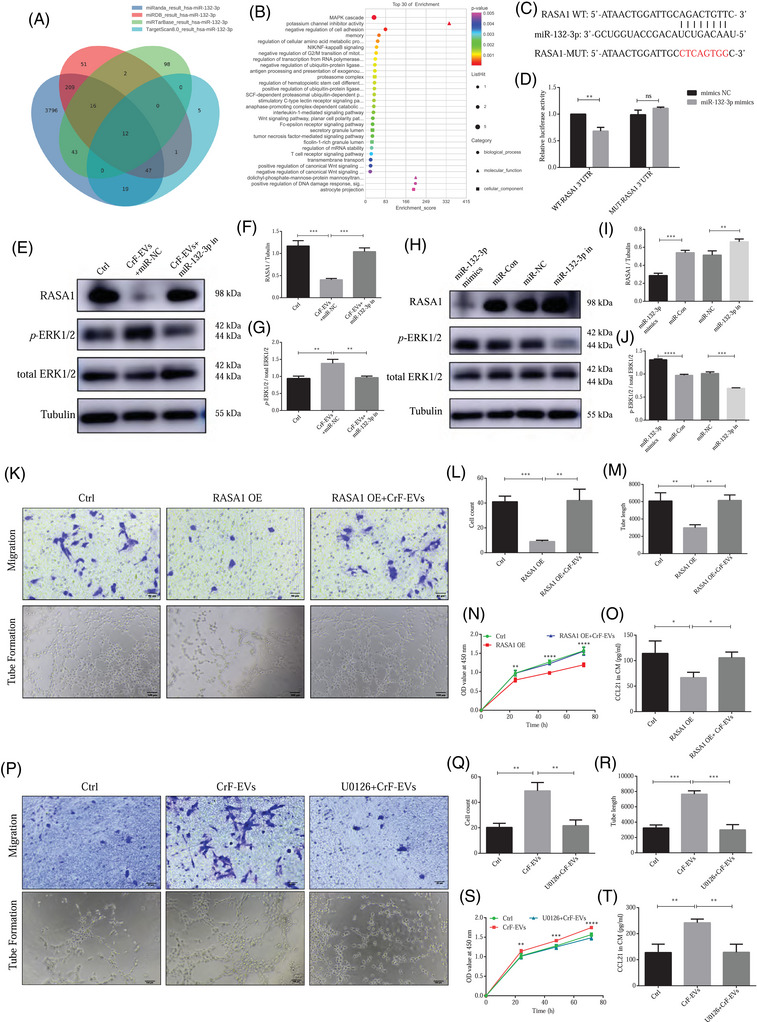
CrF‐EVs‐miR‐132‐3p promotes lymphatic endothelial functions by targeting RASA1 and activating the ERK1/2 signalling pathway. (A) Four bioinformatics databases (TargetScan, miRDB, miRTarBase and miRanda) were used to predict the downstream gene of miR‐132‐3p. (B) Gene Ontology (GO) enrichment analysis of the common 12 targets predicted in these four databases. (C) A luciferase reporter vector containing WT and mutated (MUT) binding sites within the 3′‐ UTR was constructed. (D) Luciferase activity was detected in human lymphatic endothelial cells (HLECs) treated with miR‐132‐3p mimics and normal control (NC). (E) Western blot analysis of RASA1 and *p*‐ERK1/2 expression in HLECs with miR‐132‐3p inhibition in CrF‐EVs. (F, G) Quantitative analysis of RASA1 (F) and *p*‐ERK1/2 expressions (G) in HLECs with miR‐132‐3p inhibition in CrF‐EVs. (H) RASA1 and *p*‐ERK1/2 protein expressions in HLECs with different miR‐132‐3p expressions by Western blot. (I, J) analysis of RASA1 expression (I) and *p*‐ERK1/2 activation (J) in HLECs with different miR‐132‐3p expressions. (K–M) HLECs migration and tube formation with RASA1 overexpression. (N) HLECs proliferation detected by Cell Counting Kit‐8 (CCK‐8) assay with RASA1 overexpression. (O) HLECs CCL21 secretion with RASA1 overexpression. (P–R) HLECs migration and tube formation with U0126 pre‐incubation. (S) HLECs proliferation detected by CCK‐8 assay with U0126 pre‐incubation. (T) HLECs CCL21 secretion U0126 pre‐incubation. Data are expressed as means ± SD. *p < .05, **p < .01 and ***p < .001.

To explore the molecular mechanism by which CrF‐EVs‐miR‐132‐3p modulates lymphatic endothelial function, we retrieved four publicly available bioinformatic databases (Targetscan, miRDB, miRTarBase and miRanda) for prediction of the downstream genes of miR‐132‐3p in LECs. There are a total of 12 genes predicted together in these four databases (Figure 5A), and the analysis of Gene Ontology (GO) enrichment revealed that MAPK signalling ranked at the top (Figure 5B). Among the potential predicted targets, RASA1 is an essential regulator of the MAPK pathway and effectively suppresses the signalling transduction of ERK1/2 and the downstream of the MAPK pathway.^34^, ^35^ Moreover, it plays an important role in regulating endothelial functions.^36^, ^37^ Interestingly, activation of ERK1/2 has also been proven to increase chemokine CCL21 production in endothelial cells.^38^, ^39^ In HLECs, luciferase reporter assays were used to confirm whether miR‐132‐3p could directly bind to RASA1's 3′‐UTR. Potential binding sites were constructed for both WT and MUT 3′‐UTR sequences. After miRNA‐132‐3p overexpression via miR‐132‐3p mimic, the luciferase activity was comparable in the MUT group, whereas it was significantly suppressed in the WT group (Figure 5C,D).

Of note, we found that decreased RASA1 expression after incubation of CrF‐EVs was due to decreased protein level rather than mRNA. This may be related to the post‐transcriptional regulation of miR‐132‐3p. After the inhibition of miR‐132‐3p, CrF‐EVs lost their downregulation of RASA1 in HLECs (Figure 5E–G). miR‐132‐3p mimics transfected in HLECs drastically inhibited RASA1 expression, while miR‐132‐3p inhibitor had the opposite effect (Figure 5H–J). In addition, the phosphorylation of ERK1/2 in HLECs displays a contrary alteration to RASA1 (Figure 5E–J). This was in line with the role of RASA1 as a suppressor in ERK1/2 activation.

For validation of the regulation of RASA1 in lymphatic endothelial functions, we transfected HLECs with plasmids to achieve RASA1 overexpression. With plasmid transfection, we detected that RASA1 expression was significantly elevated while ERK1/2 activation was further suppressed (Figure S8A–C). The functions of HLECs were significantly impaired with high RASA1 levels, whereas RASA1‐overexpressed HLECs incubated with CrF‐EVs displayed no functional differences compared to the control group (Figure 5K–N). ERK1/2 activation has been reported to regulate endothelial functions, and U0126 was recognized as its classical inhibitor. Our results showed that pre‐incubation with U0126 suppressed ERK1/2 activation (Figure S8D–F) and abolished the enhanced endothelial functions of HLECs stimulated by CrF‐EVs treatment (Figure 5P–S). Overexpression of RASA1 in HLECs decreased CCL21 secretion, which was abrogated by CrF‐EVs (Figure 5O). However, U0126 incubation could reverse this effect as expected (Figure 5T). Collectively, our study demonstrates that CrF‐EV‐miR‐132‐3p promotes lymphatic endothelial functions by targeting RASA1 and activating ERK1/2 signalling.

### 3.6 CrF‐EV‐miR‐132‐3p targets Rasa1 expression of LECs and promotes lymphangiogenesis and lymphatic drainage in vivo

**FIGURE 6:**
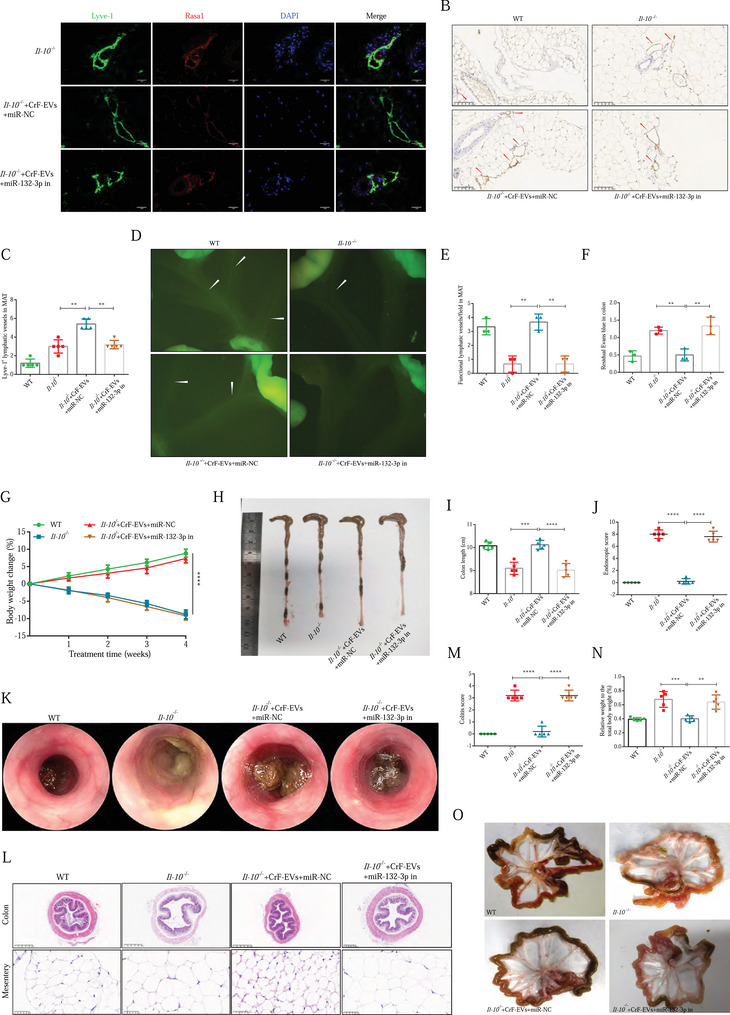
CrF‐EVs‐miR‐132‐3p targeted Rasa1 expression of human lymphatic endothelial cells (HLECs) and alleviated inflammation in the mesentery and colon of *Il‐10* ^−/–^ mice. (A) IF double staining of Lyve‐1 and Rasa1 in the mesentery of *Il‐10* ^−/−^ mice. (B, C) IHC staining of Lyve‐1 and analysis in the mesentery of *Il‐10* ^−/−^ mice. (D, E) Performance under a fluorescence microscope (D) and quantitative analysis (E) of functional lymphatic vessels after oral administration of BODITY dye. (F) Evans blue dye lymphatic drainage in the colon. (G) The weight curve of mice during the 4‐week injection of extracellular vesicles (EVs). (H) The colon length and (I) the quantitative analysis. (J) Endoscopic score of colitis of four groups. (K) The endoscopic image. (L) HE staining of the colon and mesentery. (M) Histological score of colitis. (N) The ratio of mesenteric weight to body weight. (O) The gross manifestations of mesentery. Data are expressed as means ± SD. *p < .05, **p < .01, ***p < .001, ****p < .0001 and *n* = 5 mice in each group.

We analyzed the co‐localization of Rasa1 and Lyve‐1 in LECs by IF double staining in *Il‐10* ^−/−^ mice after EVs administration. Rasa1 expression in LECs of both the mesentery and colon were significantly suppressed in *Il‐10* ^−/−^ mice after receiving CrF‐EVs, with weak Rasa1 fluorescence signal in Lyve‐1^+^ LECs (Figure 6A and Figure S9A). Meanwhile, antagonism of miR‐132‐3p rescued CrF‐EV‐induced Rasa1 suppression in the lymphatic vessels of both the mesentery and colon (Figure 6A and Figure S9A).

In line with that, *Il‐10* ^−/−^ mice receiving CrF‐EVs transfected miR‐132‐3p inhibitor exhibited similar LVD in both the mesentery and colon compared with untreated *Il‐10* ^−/−^ mice (Figure 6B,C and Figure S9B,C). Moreover, the CrF‐EVs mediated promotion of mesenteric and colonic lymphatic drainage were both reversed with miR‐132‐3p antagonism (Figure 6D–F). Therefore, our data demonstrates that the LVD and lymphatic drainage in both the mesentery and colon of *Il‐10* ^−/−^ mice were regulated by miR‐132‐3p contained in CrF‐EVs.

### 3.7 CrF‐EV‐miR‐132‐3p alleviates inflammation in the mesentery and colon of *Il‐10* ^−/–^ mice

Next, we investigated the effects of CrF‐EV‐miR‐132‐3p on the mesenteritis and colitis of *Il‐10* ^−/–^ mice. In line with our previous results, CrF‐EVs abrogated the mesenteritis and colitis of *Il‐10* ^−/–^ mice after EVs’ miR‐132‐3p was antagonized. As expected, there were no obvious differences in weight change, colon length, colitis scores and the ratio of mesenteric weight to body weight between *Il‐10* ^−/–^ mice receiving miR‐132‐3p antagonized CrF‐EVs and untreated *Il‐10* ^−/–^ mice (Figure 6G–O and Figure S10). The immune cell infiltration and inflammatory production remained unchanged in the mesentery and colon after inhibition of CrF‐EV‐miR‐132‐3p (Figure S11). Consistent with alterations in mesenteric weight and adipocyte size, the browning phenotype of mesenteric adipocytes in CrF‐EV‐treated *Il‐10* ^−/–^ mice was drastically reversed with EVs’ miR‐132‐3p inhibition (Figure S12). Thus, our data indicated that CrF‐EV‐miR‐132‐3p boosts lymphangiogenesis and enhances lymphatic drainage function in the colon and MAT, which further attenuates spontaneous mesenteritis and colitis of *Il‐10* ^−/–^ mice.

### 3.8 LVD and inflammation in patients with CD are positively associated with miR‐132‐3p expression

**FIGURE 7:**
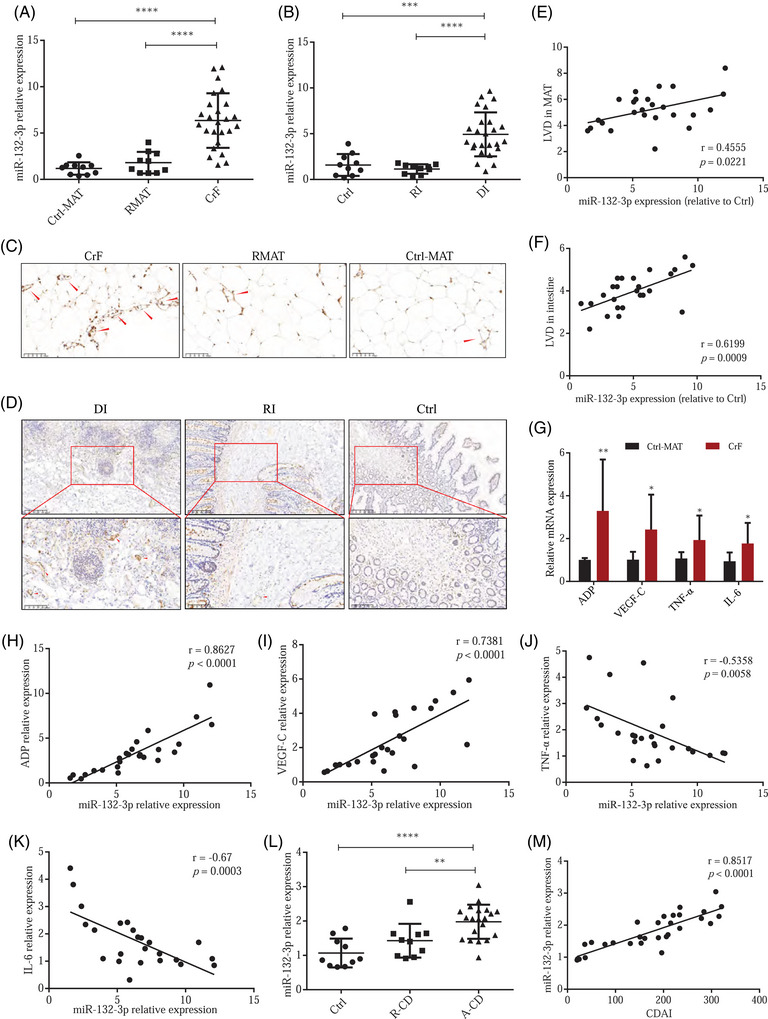
Elevated expression of miR‐132‐3p was tightly associated with lymphatic vessel density (LVD) and inflammation in Crohn's disease (CD) patients. (A) Expressions of miR‐132‐3p in CrF, resection margin of MAT (RMAT) and non‐CD MAT (Ctrl‐MAT). (B) miR‐132‐3p level in the inflammatory intestine (DI), resection margin of the intestine (RI) and non‐CD intestine (Ctrl). (C) IHC of LYVE‐1 in CrF, RMAT and Ctrl‐MAT. (D) LYVE‐1 staining by IHC in intestine samples. (E, F) Spearman correlation analysis of miR‐132‐3p and LVD in mesentery (E) and intestine (F), respectively. (G) Quantitative polymerase chain reaction (qPCR) detected expression of ADP, VEGF‐C, TNF‐α and IL‐6 in CrF and Ctrl‐MAT samples. (H–K) Spearman correlation analysis of miR‐132‐3p and ADP (H), VEGF‐C (I), TNF‐α (J) and IL‐6 (K), respectively. (L) qPCR detected miR‐132‐3p expression in the serum of healthy control (Ctrl), active CD patients (A‐CD) and remission CD patients (R‐CD) patients. (M)Spearman correlation analysis of serum miR‐132‐3p and CDAI in CD patients. Data are expressed as means ± SD. *p < .05, **p < .01, ***p < .001 and ****p < .0001.

We evaluated the expression of miR‐132‐3p in the intestinal and mesenteric samples from 25 CD patients and 10 non‐CD patients to validate our results. We found that the diseased intestine and the corresponding mesentery (CrF) displayed significantly elevated miR‐132‐3p levels compared to the resections of margin mesentery and intestine tissues (RMAT and RI) as well as to the control samples (Ctrl and Ctrl‐MAT) (Figure 7A,B). Furthermore, IHC staining (Figure 7C,D and Figure S13A,B) showed that the inflammatory intestine and the corresponding mesentery (CrF) exhibited significantly increased LVD than in the margin tissues and control samples. A positive correlation was found between the miR‐132‐3p level in the mesentery (*r *= 0.4555, *p = *.0221, Figure 7E) and the intestine (*r *= 0.6199, *p = *.0009, Figure 7F). Interestingly, we detected elevated CCL21 expression in CrF compared with RMAT and Ctrl‐MAT samples, which was mainly in LECs (Figure S13C). The abnormal expression of adipokines and cytokines in CrF is a key pathological change involved in CD progression. In line with the previous study,^5^ our results of qPCR showed significantly increased levels of adiponectin (ADP), TNF‐α and IL‐6 in CrF (Figure 7G). Notably, VEGF‐C, the essential factor for lymphangiogenesis, was also significantly elevated in CrF (Figure 7G). Correlation analysis further showed that miR‐132‐3p was positively related with ADP and VEGF‐C, while negatively with TNF‐α and IL‐6 (*r *= 0.8627, *p <* .0001 for ADP; *r *= 0.7381, *p <* .0001 for VEGF‐C; *r *= ‐0.5358, *p = *.0058 for TNF‐α; *r *= ‐0.67, *p = *.0003 for IL‐6, Figure 7H–K). Collectively, it appears that the miR‐132‐3p elevation in CrF may be influenced by VEGF‐C and ADP and may have active lymphatics‐stimulating and anti‐inflammatory activities, which is consistent with our conclusions in the current experiments.

Furthermore, in order to determine the value of miR‐132‐p as a biomarker for CD diagnosis or prognosis, we investigated the level of serum miR‐132‐3p and its correlation with CD progression. As shown in Figure 7L, active CD patients (*n* = 20) exhibited significantly higher levels of serum miR‐132‐3p as compared to inactive CD patients (*n* = 10) or healthy controls (*n* = 10). Moreover, serum miR‐132‐3p expression was positively correlated with Crohn's disease activity index (CDAI, *r *= 0.8517, *p <* .0001, Figure 7M), showing its close participation in CD activity. Taken together, this data highlights the potential for miR‐132‐3p to be used as a biomarker for monitoring disease progress and severity, with the benefit of being obtained via minimally invasive serum sampling.

Based on these findings, we have demonstrated that CrF‐EVs have significant beneficial roles in improving lymphatic functions and alleviating colitis and mesenteritis via the miR‐132‐3p/RASA1/ERK1/2 axis.

## 4 DISCUSSION

CrF, a hallmark of CD, refers to hypertrophic MAT wrapping around intestinal lesions. Although its influence on CD is still debated within the field, the most widely accepted viewpoint is that CrF plays a protective role in disease progression.^3^, ^4^, ^5^ ADSCs are an essential cellular component of the interstitial cells in adipose tissue and regulate inflammatory diseases via EV secretion and delivery. Although several studies have shown the abnormal characteristics of stem cells from IBD‐derived ADSCs,^40^, ^41^ the exact effects of CrF‐derived ADSCs on intestinal and mesenteric lesions in CD remain unknown. In this study, we revealed that CrF‐ADSCs produce EVs to attenuate the inflammation of the colon and mesentery. Mechanistically, CrF‐EVs mitigate colitis and mesenteritis by delivering miR‐132‐3p to LECs and targeting the RASA1/ERK1/2 axis, which promotes lymphangiogenesis and improves lymphatic drainage function.

Lymphatic vessels play an important role in the drainage of lymph, lipids and immune cells.^41^ Interestingly, LECs are the main resource of CCL21, the crucial chemokine which binds to its receptor CCR7 on the surface of immune cells. Via CCL21‐CCR7 interaction, immune cells emigrate from inflamed tissues leading to inflammation alleviation.^42^, ^43^ CD has long been recognized as a disease with dysfunctions in the lymphatic system. The lymphatic vessels in the intestine and mesentery of CD patients have been shown to be discontinuous and highly permeable, causing lymph leakage and drainage dysfunction.^15^ All these pathological changes lead to lymphatic stasis and lymphedema of tissues, which further exacerbates chronic inflammation, fibrosis and adipose hyperplasia of the mesentery.^13^ Approaches focusing on the stimulation of LVD and the improvement of lymphatic function have achieved satisfactory outcomes in CD treatment. ADSCs pre‐treated with VEGF‐C effectively enhance lymphatic vessel drainage and alleviate intestinal inflammation.^18^ Furthermore, blockade of VEGF‐C/VEGFR‐3 signalling, the molecular mechanism of lymphangiogenesis, leads to lymphatic vessel enlargement and aggravated experimental colitis.^44^

*Il10* ^−/−^ mice, a classic animal model of spontaneous colitis and mesenteritis, mimics human CD and displays overt lymphatic vessel dysfunction and mesenteric hypertrophy.^28^ It was reported that adipokine apelin could ameliorate chronic colitis in *Il10* ^−/−^ mice by promoting intestinal lymphatic function.^23^ In addition, chylomicrons‐simulating sustained drug release in mesenteric lymphatics effectively mitigated Crohn‐like colitis in *Il10* ^−/−^ mice.^25^ Of note, CrF and its surrounding intestinal tract display significantly elevated LVD and are tightly related to CD recurrence and prognosis.^17^, ^21^ Considering the protective role of CrF, we inferred that the ADSCs from CrF secret EVs to regulate lymphatic vessel function and density, which may be another compensatory mechanism of CD progression.

Nowadays, scientific research is increasingly focused on the ADSCs from IBD patients. ADSCs derived from UC and CD patients have been reported to exhibit aberrant capabilities of proliferation and adipogenic differentiation,^45^ and there is a distinct DNA methylation pattern in subcutaneous ADSCs of CD.^46^ Nevertheless, these studies mainly focus on their stemness phenotypes in vitro. According to recent studies, the anti‐inflammatory abilities of ADSCs are generally enhanced by an abnormal microenvironment, such as hypoxia or inflammation.^47^, ^48^ Additionally, CrF‐ADSCs exhibit a strong potential for brown differentiation, which has already been demonstrated to be an anti‐inflammatory mechanism in CD MAT.^5^, ^9^ In a recent study,^12^ CrF‐derived ADSCs promoted fibroblast activation and collagen deposition in intestinal fibrosis. However, due to a lack of animal models, the influences of CrF‐ADSCs on colitis and mesenteritis of CD remain elusive. In our study, *Il‐10* ^−/–^ mice exhibited overt intestinal lesions and mesenteric hypertrophy from the age of 15 weeks old. We observed that a 4‐week intervention of CrF‐ADSCs EVs effectively mitigated disease progression in the colon and mesentery of *Il‐10* ^−/–^ mice.

EVs are produced and secreted from host cells and transport essential cargo, including miRNAs, proteins and metabolites. ADSCs have been reported to interact and regulate other cell types mainly through EVs, within which miRNAs play an essential role. In order to investigate the specific mechanisms of cellular communication, we used miRNA high‐throughput sequencing and found that miR‐132‐3p was highly expressed by CrF‐ADSCs and their EVs, which could be delivered to LECs. Our previous study^26^ has demonstrated that ADSCs from subcutaneous adipose secreted EVs’ miR‐132 and promoted LEC functions in vitro. In line with that, this study exemplified that CrF‐EVs promoted lymphangiogenesis and improved lymphatic endothelial functions. Moreover, these influences were both abrogated by EVs’ miR‐132‐3p antagonism.

Using bioinformatic databases, we predicted 12 potential targets which were highly enriched in MAPK signal transduction. RASA1 is the regulator of ERK1/2 phosphorylation that has been reported as the downstream signal of the MAPK pathway. In addition, it is reported that miR‐132‐3p targets RASA1 in the vascular endothelium and promotes angiogenesis in cerebral apoplexy.^49^ ERK1/2 activation can effectively enhance LEC proliferation, migration and tube formation.^50^ Moreover, MAPK signal transduction by ERK1/2 activation has been proven to enhance CCL21 production in LECs, which is responsible for immune cell emigration from lymphatic vessels via CCL21/CCR7 interaction.^41^, ^42^ In line with this, our study showed that CrF‐EVs delivered miR‐132‐3p to LECs and improved lymphatic functions via the RASA1/ERK1/2 axis, and also suppressed Rasa1 expression in colonic and mesenteric lymphatic vessels of *Il10^−/−^* mice. Similarly, the effects of CrF‐EVs could be abrogated with the antagonism of EVs’ miR‐132‐3p. Therefore, CrF‐EV injection regulated colitis and mesenteritis of *Il‐10* ^−/−^ mice through miR‐132‐3p/RASA1/ERK1/2‐dependent improvement of lymphatic endothelial functions.

Of course, the production of CrF‐EVs for therapeutic use faces many challenges, such as strict ethical approval, inefficient production technology and unsatisfactory delivery efficiency. Nonetheless, innovative bioengineering technologies continue to provide opportunities for clinical translational application. Recently, EVs or bioengineered EV‐like liposomes have been widely used as a delivery system for drugs or molecules. By decorating their outer surfaces with specific molecules such as peptides or antibody fragments that recognize and target antigens,^51^, ^52^, ^53^ they have demonstrated precise targeting capabilities. As previously reported, mesoporous silica nanoparticles were used as a nucleus vector for targeting lymphatics and drug transportation.^24^ A future direction could be the construction of a biological delivery system loaded with miR‐132‐3p and targeting LV, which we believe to have great clinical potential in CD treatment.

Recently, miR‐132 has been reported to be upregulated in IBD patients.^54^ Notably, higher levels of miR‐132 were present in inflamed intestinal biopsies compared to those of apparently quiescent patients of IBD, and miR‐132 targeted acetylcholinesterase to further regulate intestinal inflammation.^55^ In addition, increased expression of miR‐132 suppressed colonic inflammation, suggesting it might be a promising therapeutic candidate for restricting autoimmune inflammation.^56^ In the present study, upregulated levels of miR‐132‐3p in CrF and its surrounding intestine were detected in CD patients, which were positively correlated with increased LVD. Moreover, miR‐132‐3p was positively linked to ADP and VEGF‐C while negatively associated with IL‐6 and TNF‐α in CrF. Lymphangiogenesis is largely governed by VEGF‐C and it has been reported to stimulate stem cells of adipose tissue to increase secretion of exosomal miR‐132.^26^ ADP contributes significantly to VEGF‐C‐dependent enhancement of lymphangiogenesis and lymphatic functions.^57^ It appears that the miR‐132‐3p elevation in CrF has active lymphatics‐stimulating and anti‐inflammatory functions. Interestingly, as previously reported,^56^, ^58^ miR‐132‐3p was highly expressed in brown adipose tissue‐derived EVs, as well as lymphatic vessels that were also linked with adipose browning. In line with that, our results demonstrated that CrF‐EVs‐miR‐132‐3p could induce MAT browning through its regulation of lymphatic vessel function. Moreover, serum miR‐132‐3p level was also elevated in patients diagnosed with CD, and positively linked with disease activity, further validating its essential role in CD progression and potential value for disease assessment.

Nevertheless, our study has its limitations. First, the microenvironment of CrF is extremely complex, rich in lipids, and highly inflammatory. Therefore, it is difficult to ascertain the specific reasons for the alterations seen in CrF‐ADSCs and their EVs. Next, although enhanced CCL21 secretion from LECs was stimulated by CrF‐EVs, whether this leads to a migration of immune cells out of lymphatic vessels remains unclear, which will be our future direction. In addition, the downstream targets of miR‐132‐3p/RASA1/ERK1/2 responsible for lymphangiogenesis have not been identified in the present study. By resorting to recent references,^59^, ^60^ a preliminary hypothesis is that activator protein‐1, a transcription factor that participates in lymphatic lesions induced by oxidative stress or hypoxia, could possibly function downstream of ERK1/2 and further regulate lymphatic functions. Nevertheless, more experimental evidence is needed. However, these limitations do not undermine the significance of our study. We have provided extensive evidence showing that miR‐132‐3p plays a central role in regulating lymphatic function.

## 5 CONCLUSION

CrF‐EVs improve lymphatic function and alleviate inflammation in the colon and mesentery through the miR‐132‐3p/RASA1/ERK1/2 axis. Our work highlights a novel role of lymphatics in the progression of CD, offering new insights for its treatment.

## AUTHOR CONTRIBUTIONS

Xiaolei Wang designed the study and critically revised the whole manuscript. Xin Su and Alexandra Bartolomucci revised the whole manuscript and participated in the study design. Weigang Shu and Yongheng Wang were involved in the acquisition, analysis and interpretation of data, as well as the drafting of the manuscript and the drawing of all the figures. Chunqiu Chen, Fangtao Wang and Peng Du were involved in the acquisition and analysis of data for the work. All authors agree with the final approval of the version to be published.

## CONFLICT OF INTEREST STATEMENT

The authors declare no conflict of interest.

## ETHICS STATEMENT

This study was approved by the Institutional Ethics Committee of Shanghai Tenth People's Hospital (SHSY‐IEC‐4.1/20‐152/01 and SHDSYY‐2019‐3886).

## Supporting information



Supporting Information

Supporting Information

Supporting Information

Supporting Information

Supporting Information

Supporting Information

Supporting Information

Supporting Information

Supporting Information

Supporting Information

Supporting Information

Supporting Information

Supporting Information

Supporting Information

## Data Availability

Data are available from the corresponding author upon reasonable request.
