# Supplementary figures and images for: Extracellular vesicles derived from creeping fat stem cells promote lymphatic function and restrain inflammation of Crohn's disease
Source: Clin Transl Med. 2024 Dec 2;14(12):e70086. doi: 10.1002/ctm2.70086 (PMC11612264; doi:10.1002/ctm2.70086)

A

Colon

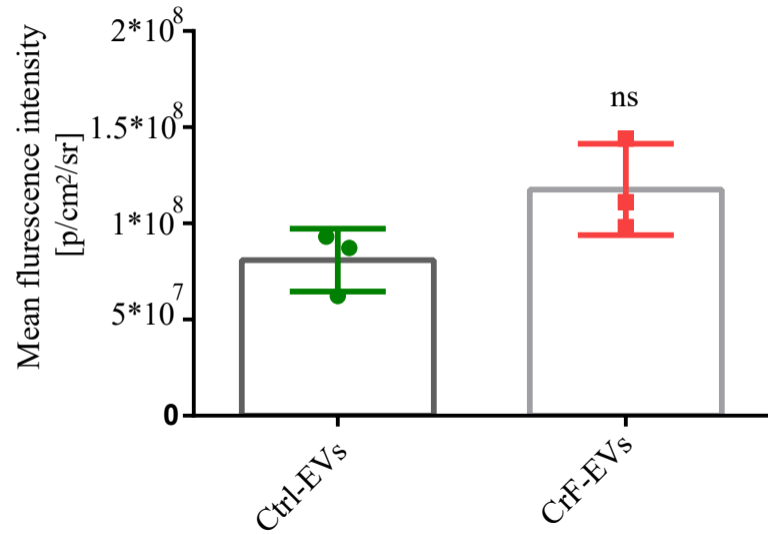

B

Mesentery

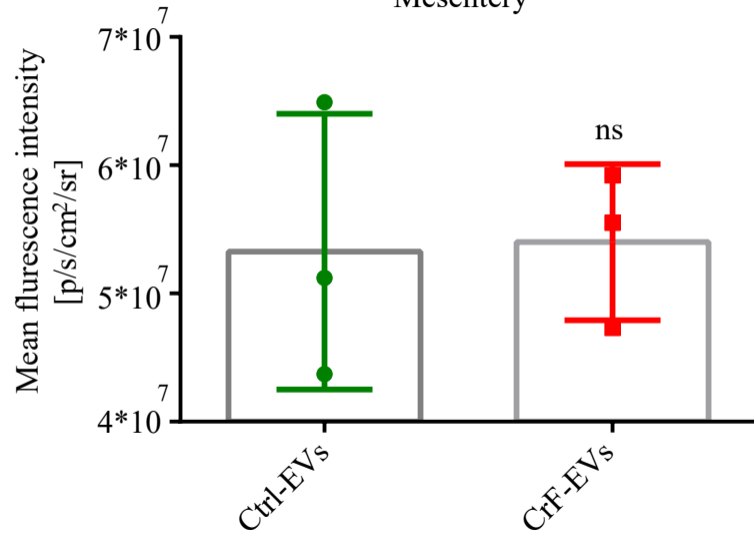

Supplement: Supplementary file 1 — Supporting Information [file CTM2-14-e70086-s013.pdf]

A

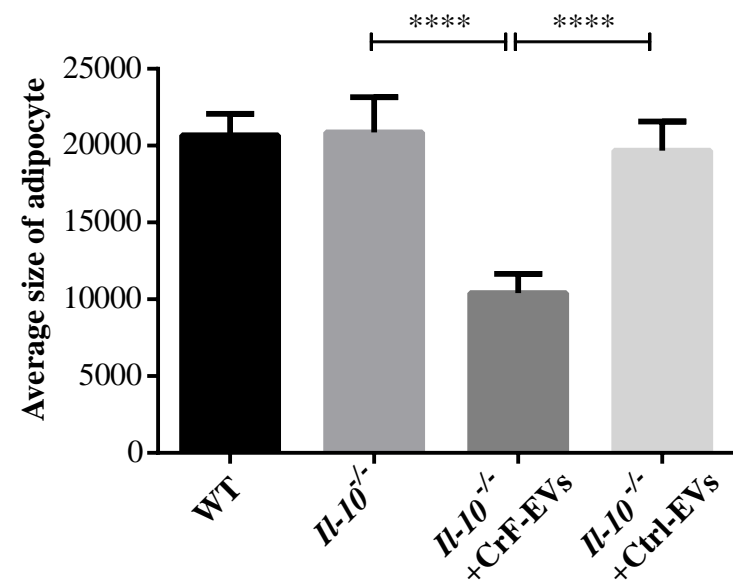

B

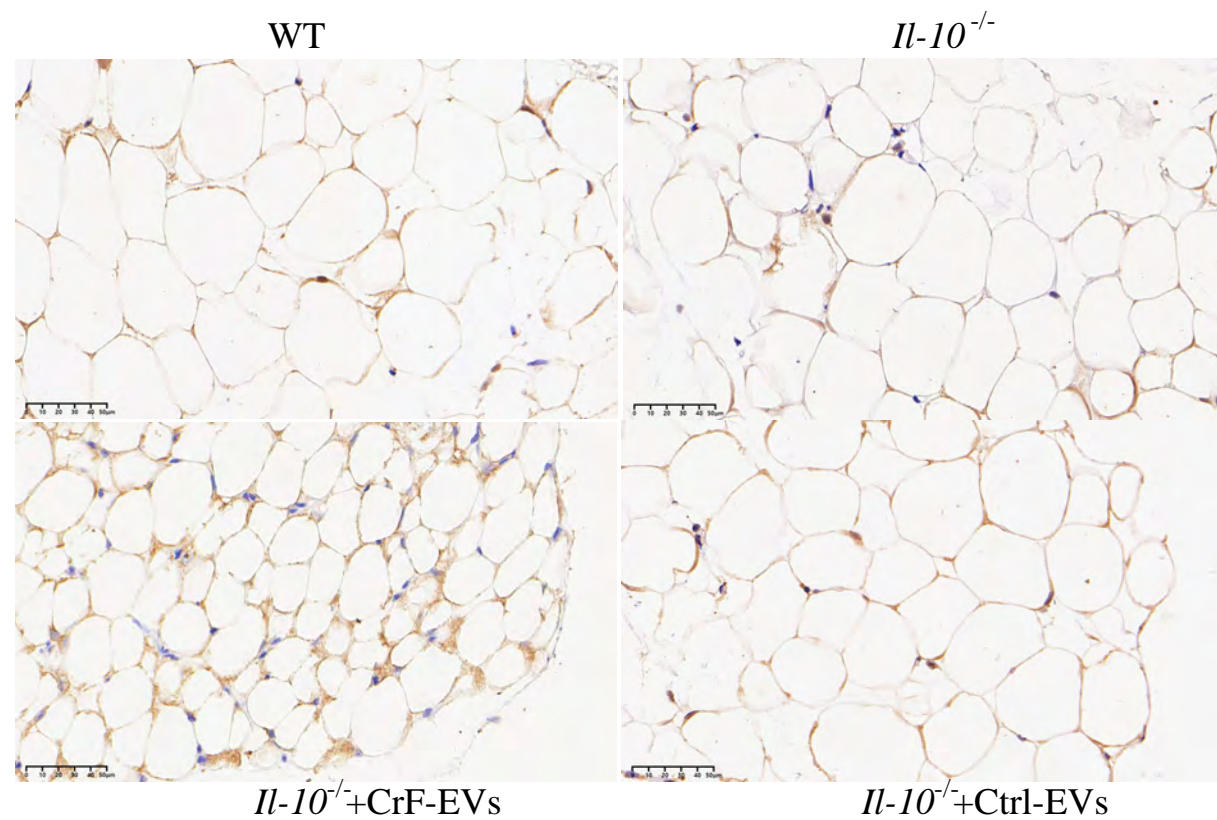

C

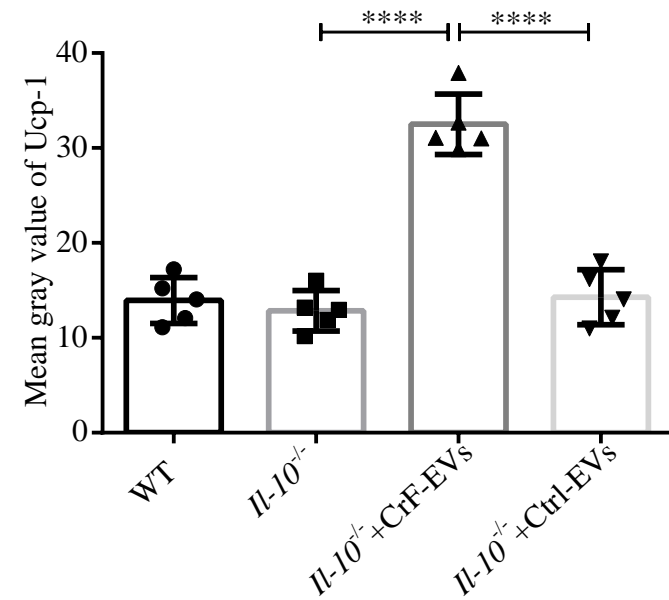

Supplement: Supplementary file 2 — Supporting Information [file CTM2-14-e70086-s008.pdf]

A

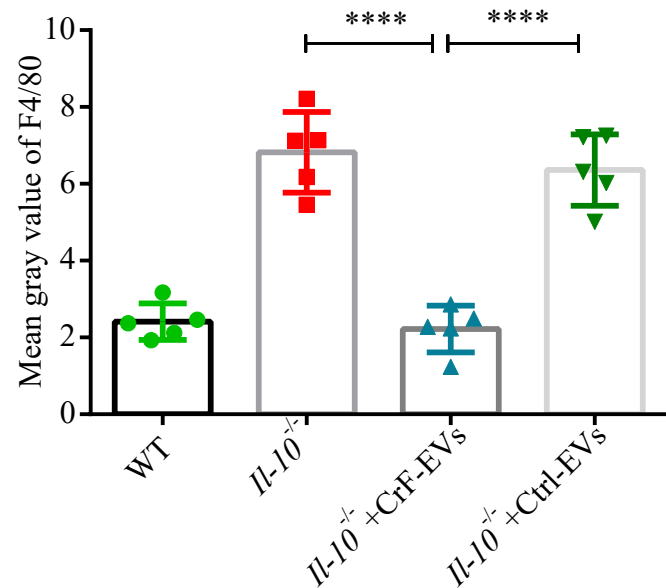

B

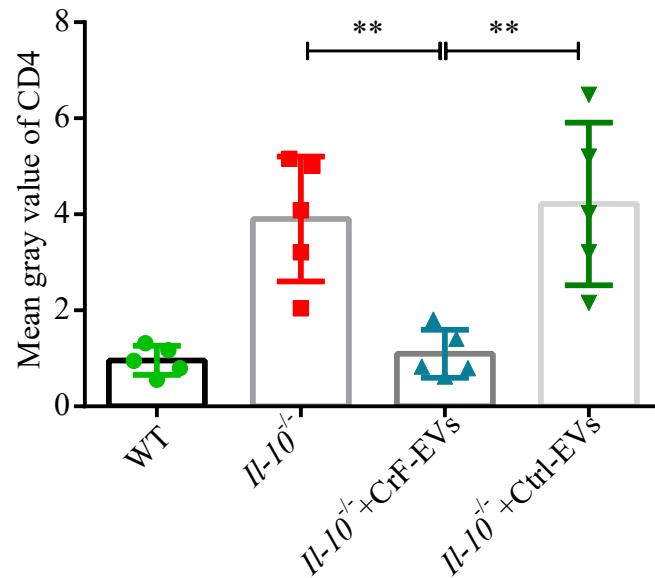

C

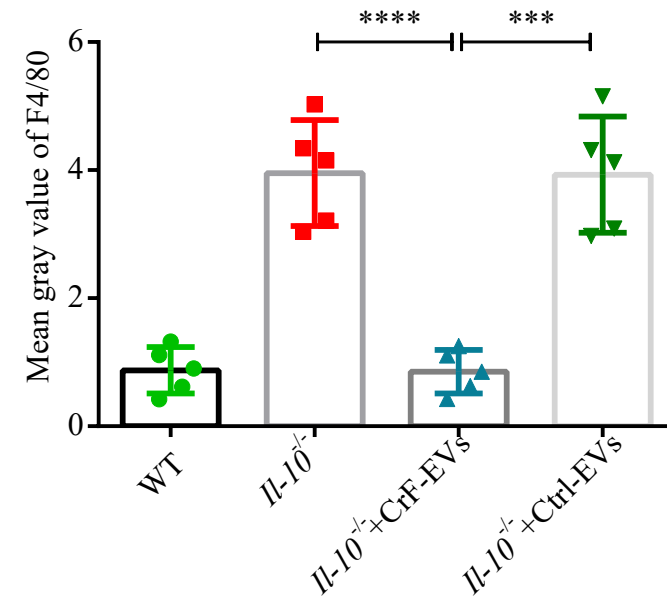

D

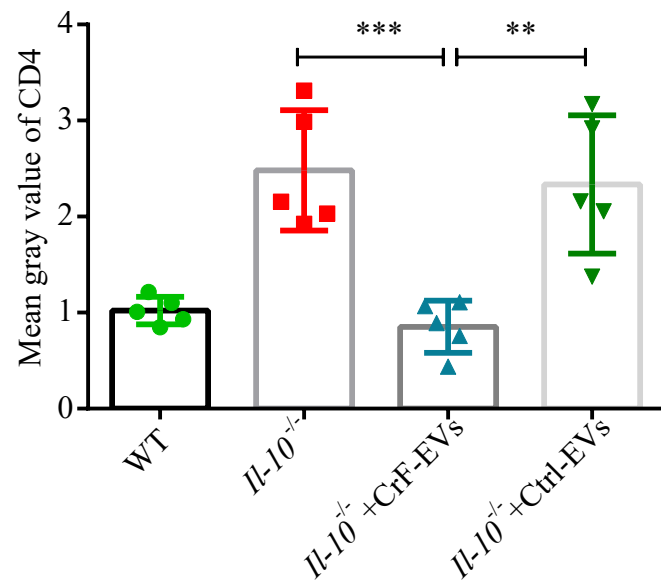

E

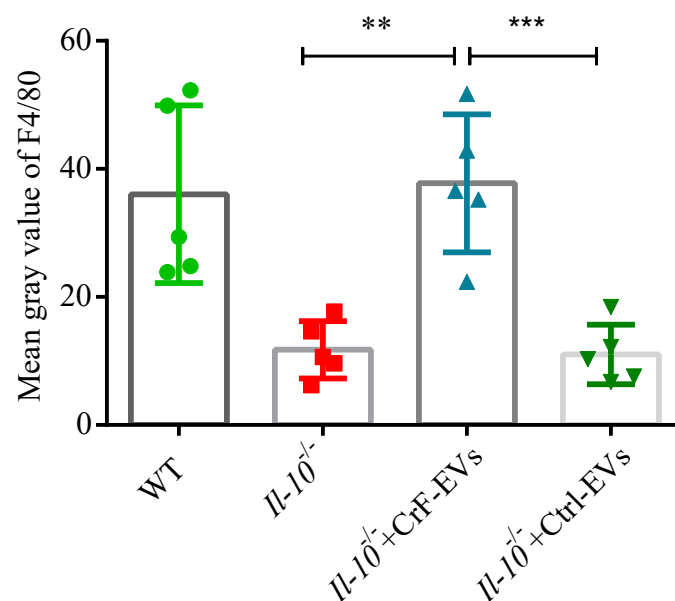

F

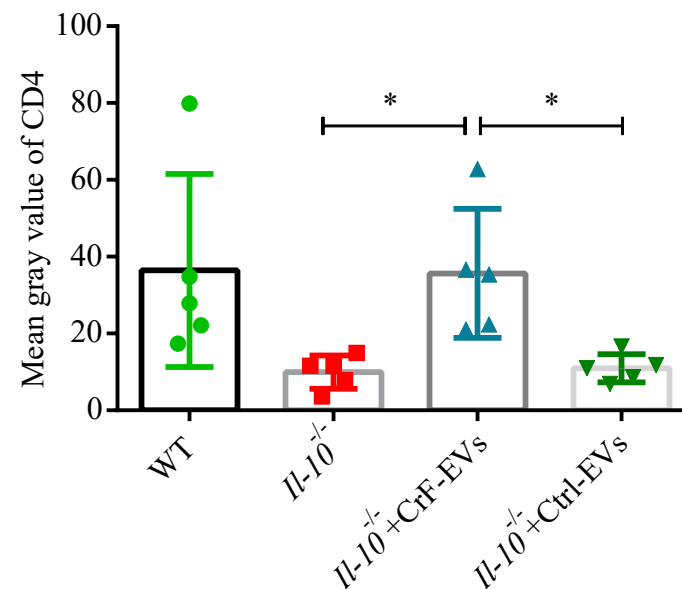

Supplement: Supplementary file 3 — Supporting Information [file CTM2-14-e70086-s009.pdf]

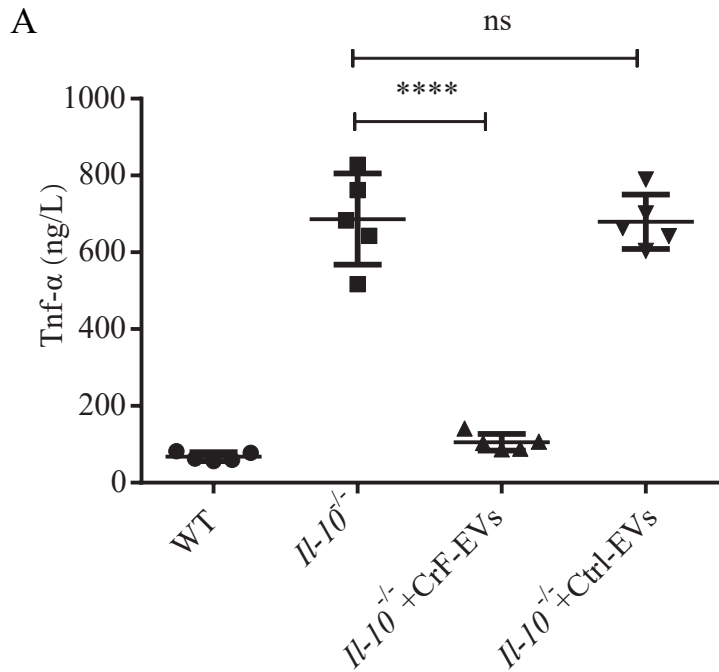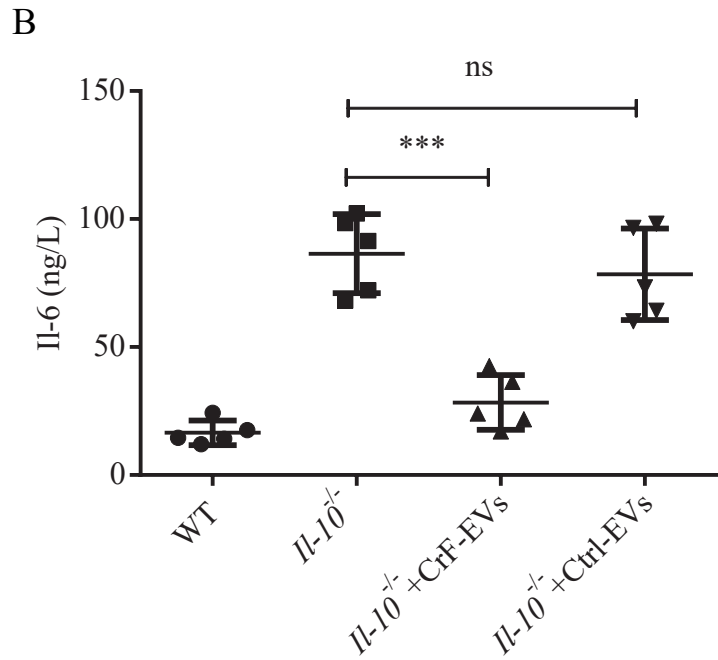

Supplement: Supplementary file 4 — Supporting Information [file CTM2-14-e70086-s010.pdf]

A

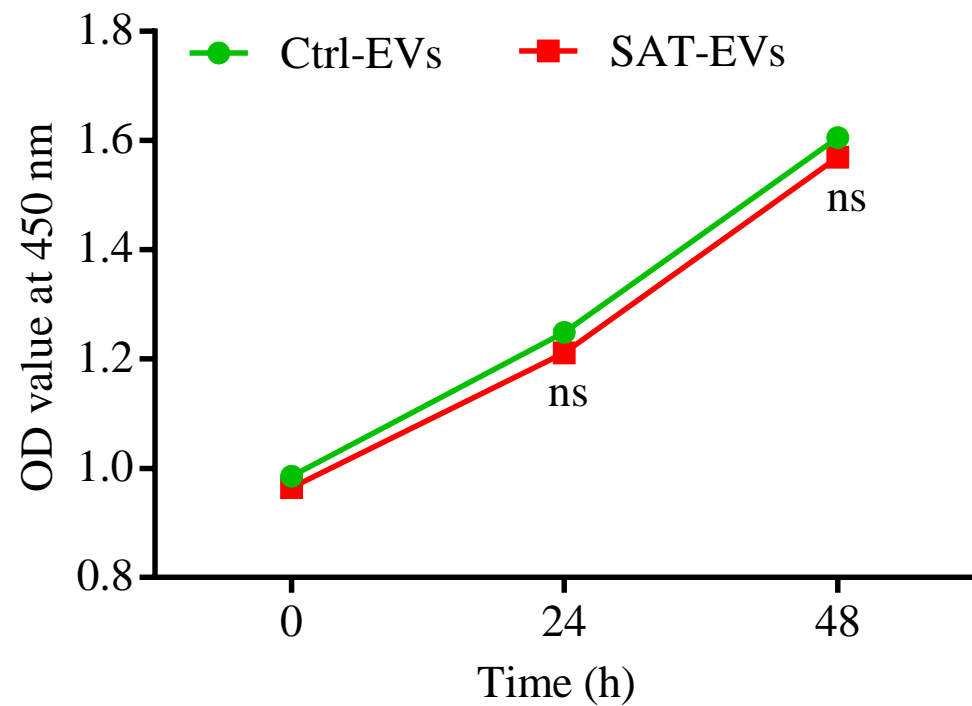

B

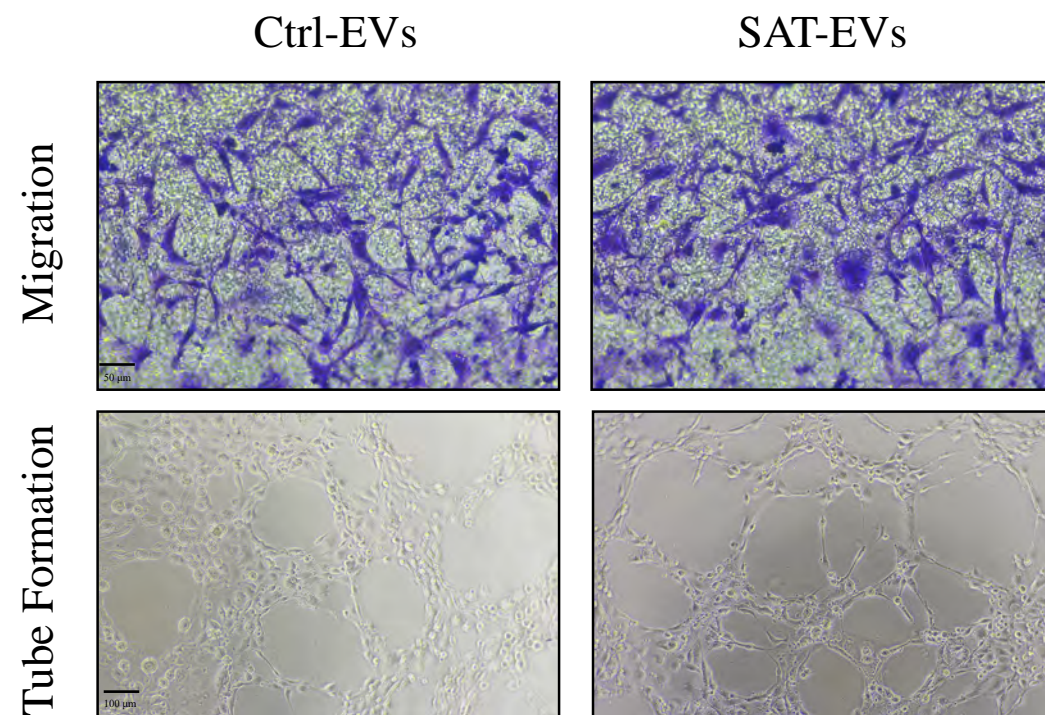

C

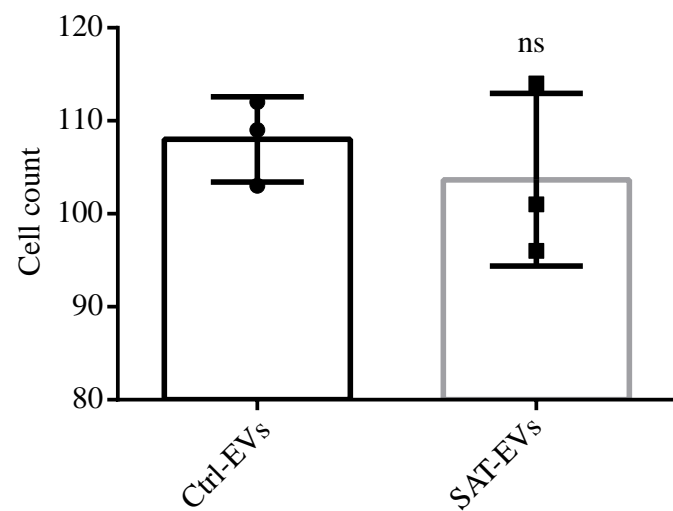

D

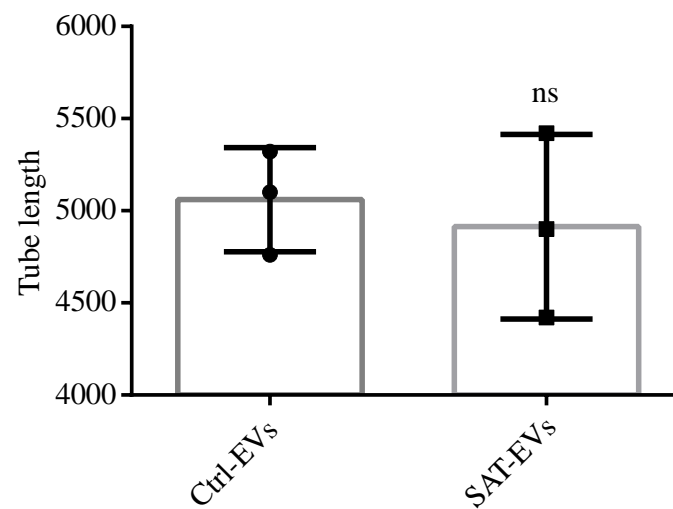

E

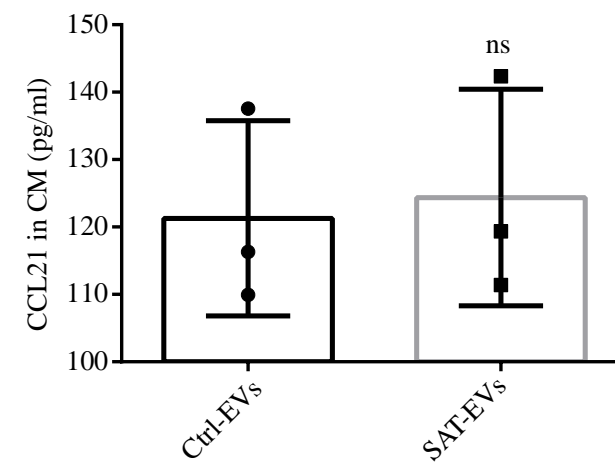

Supplement: Supplementary file 5 — Supporting Information [file CTM2-14-e70086-s014.pdf]

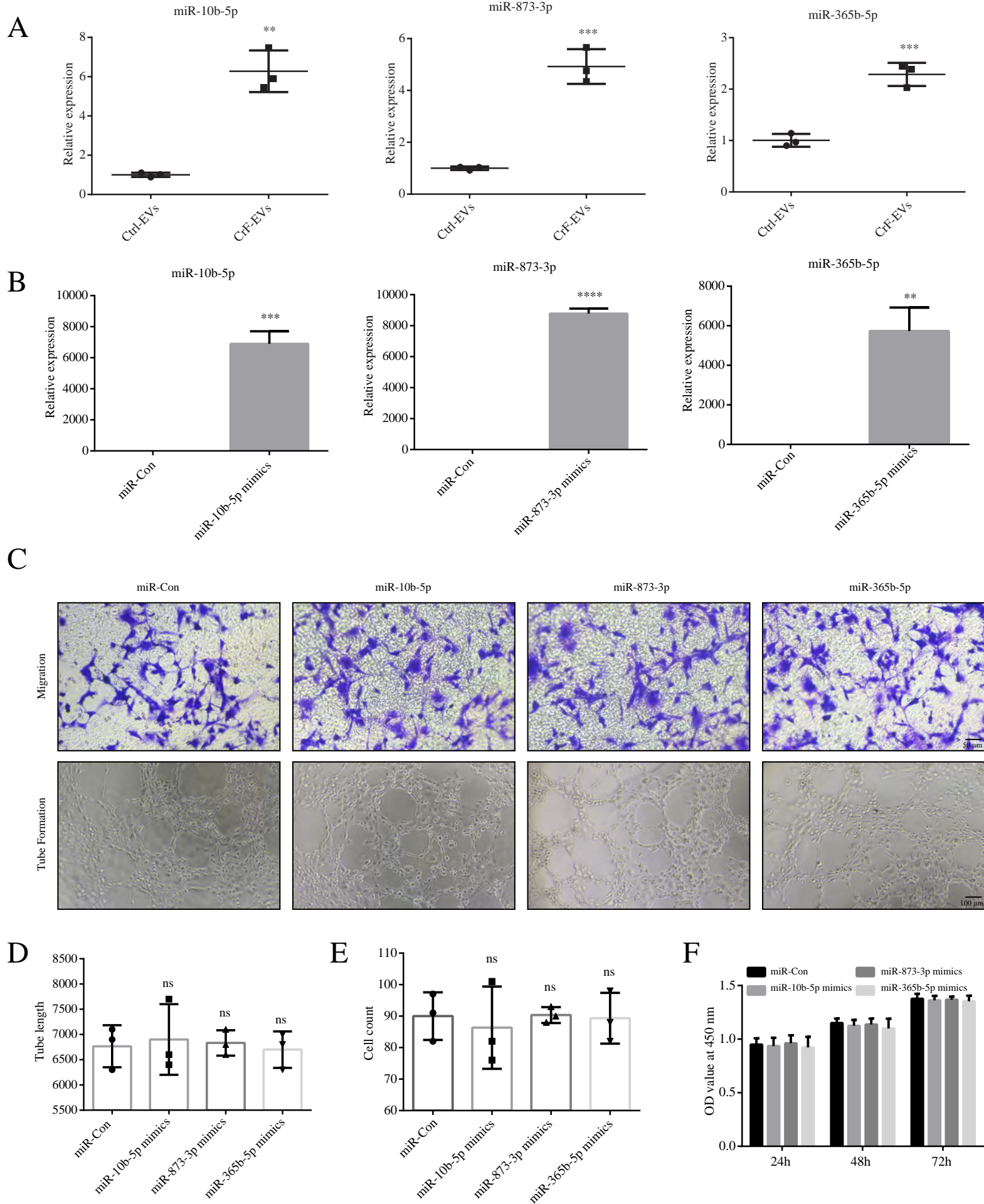

Supplement: Supplementary file 6 — Supporting Information [file CTM2-14-e70086-s003.pdf]

A

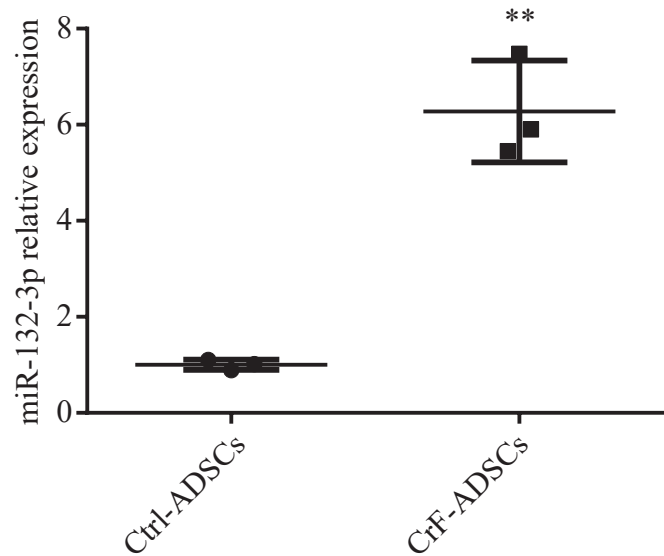

B

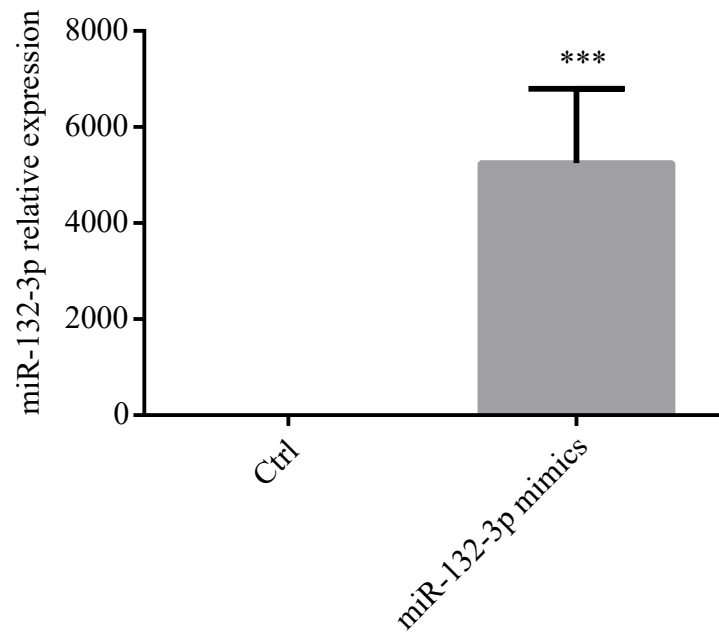

Supplement: Supplementary file 7 — Supporting Information [file CTM2-14-e70086-s012.pdf]

A

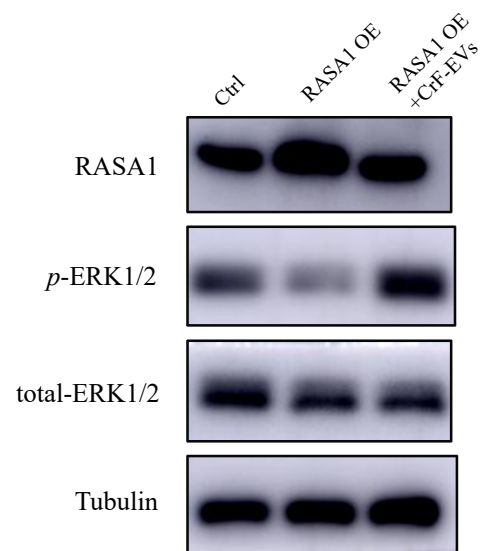

B

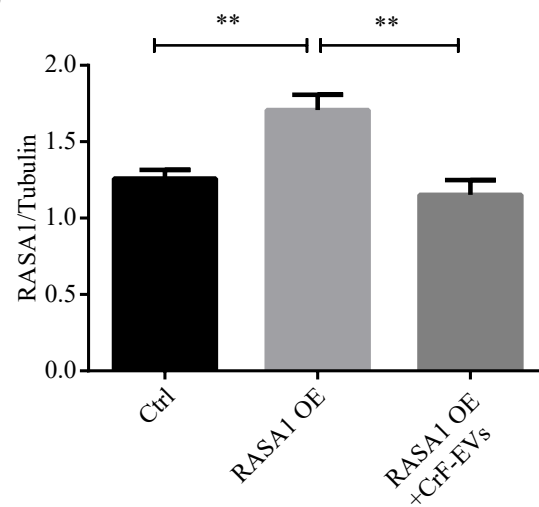

C

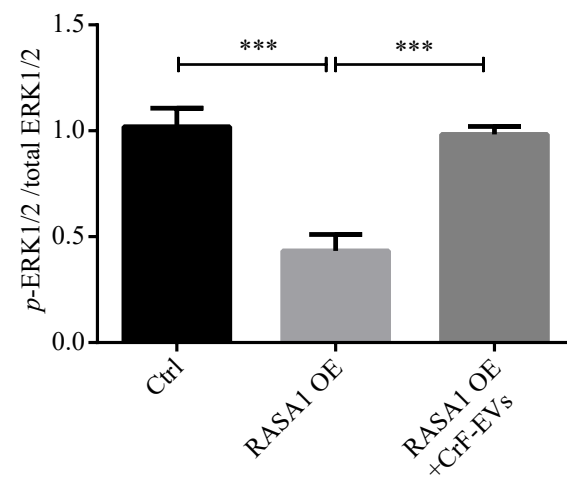

D

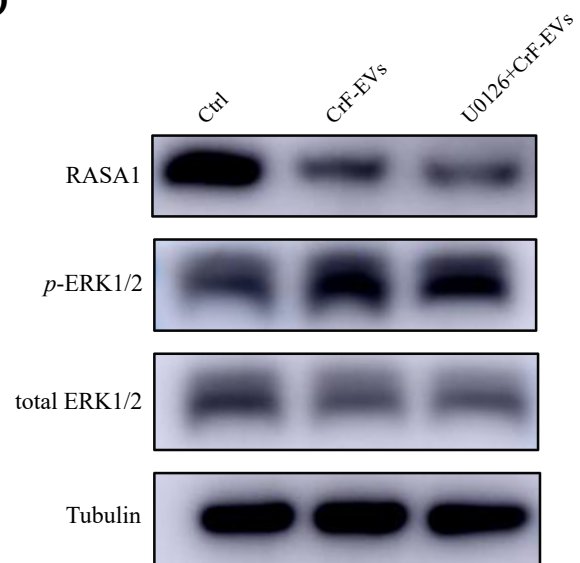

E

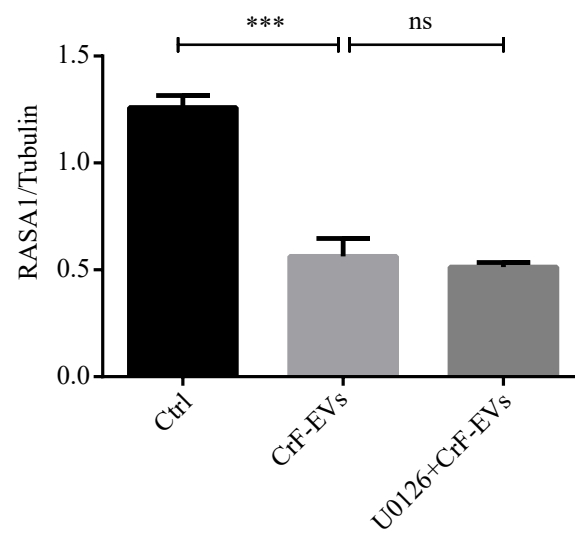

F

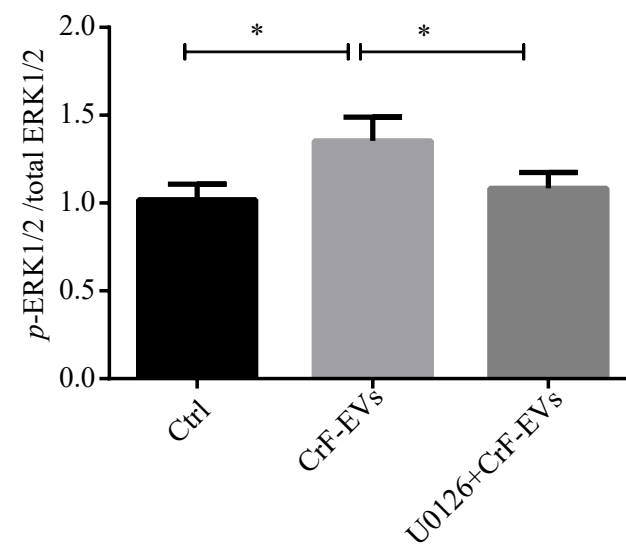

Supplement: Supplementary file 8 — Supporting Information [file CTM2-14-e70086-s005.pdf]

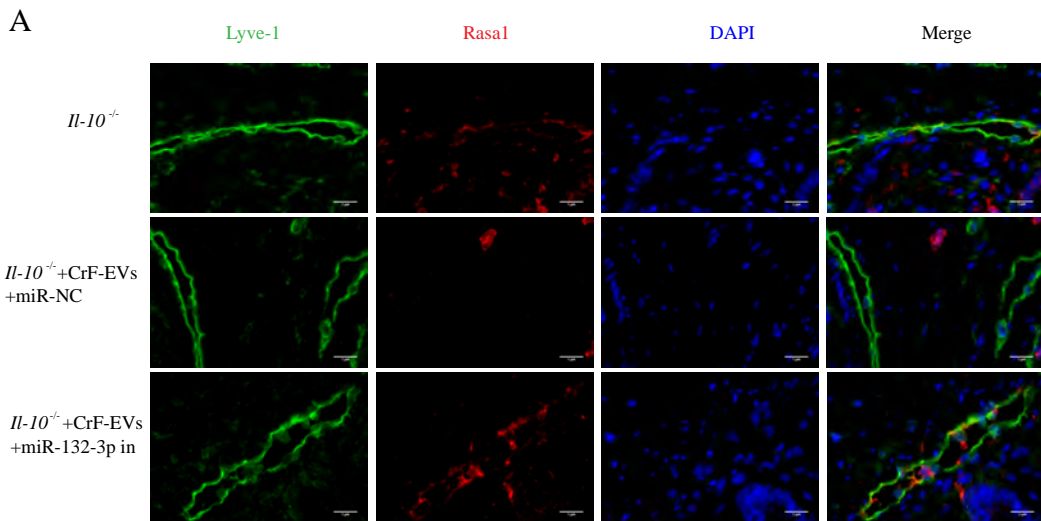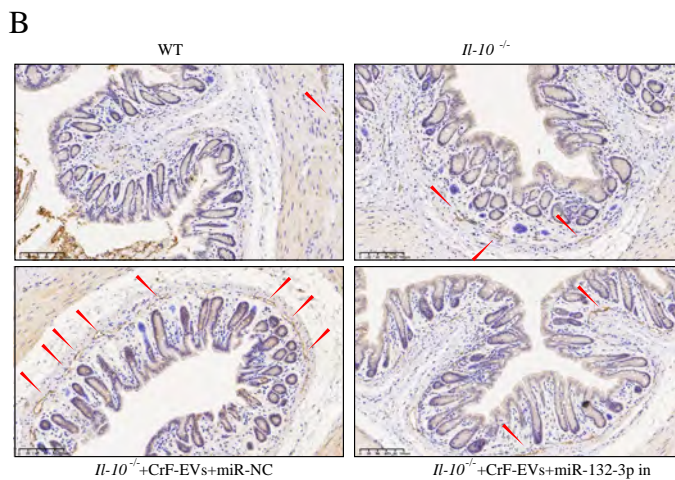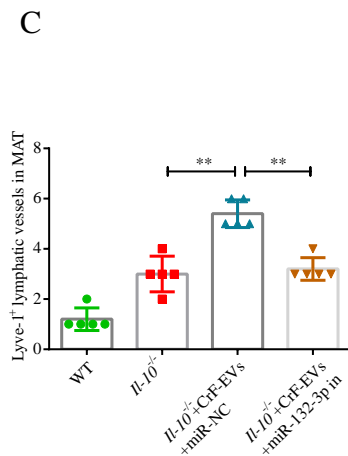

Supplement: Supplementary file 9 — Supporting Information [file CTM2-14-e70086-s001.pdf]

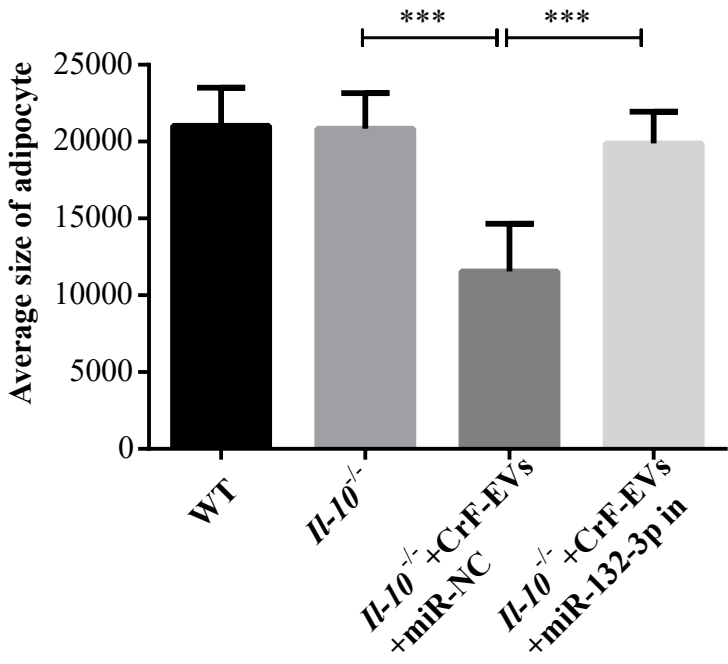

Supplement: Supplementary file 10 — Supporting Information [file CTM2-14-e70086-s011.pdf]

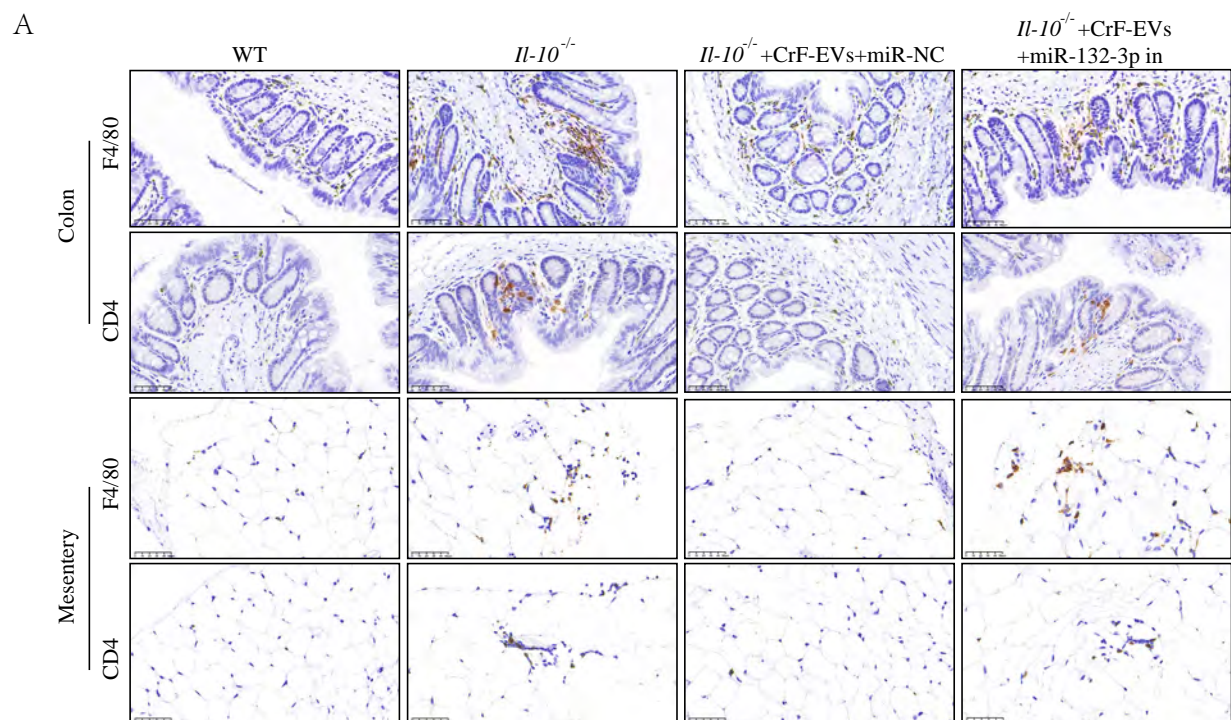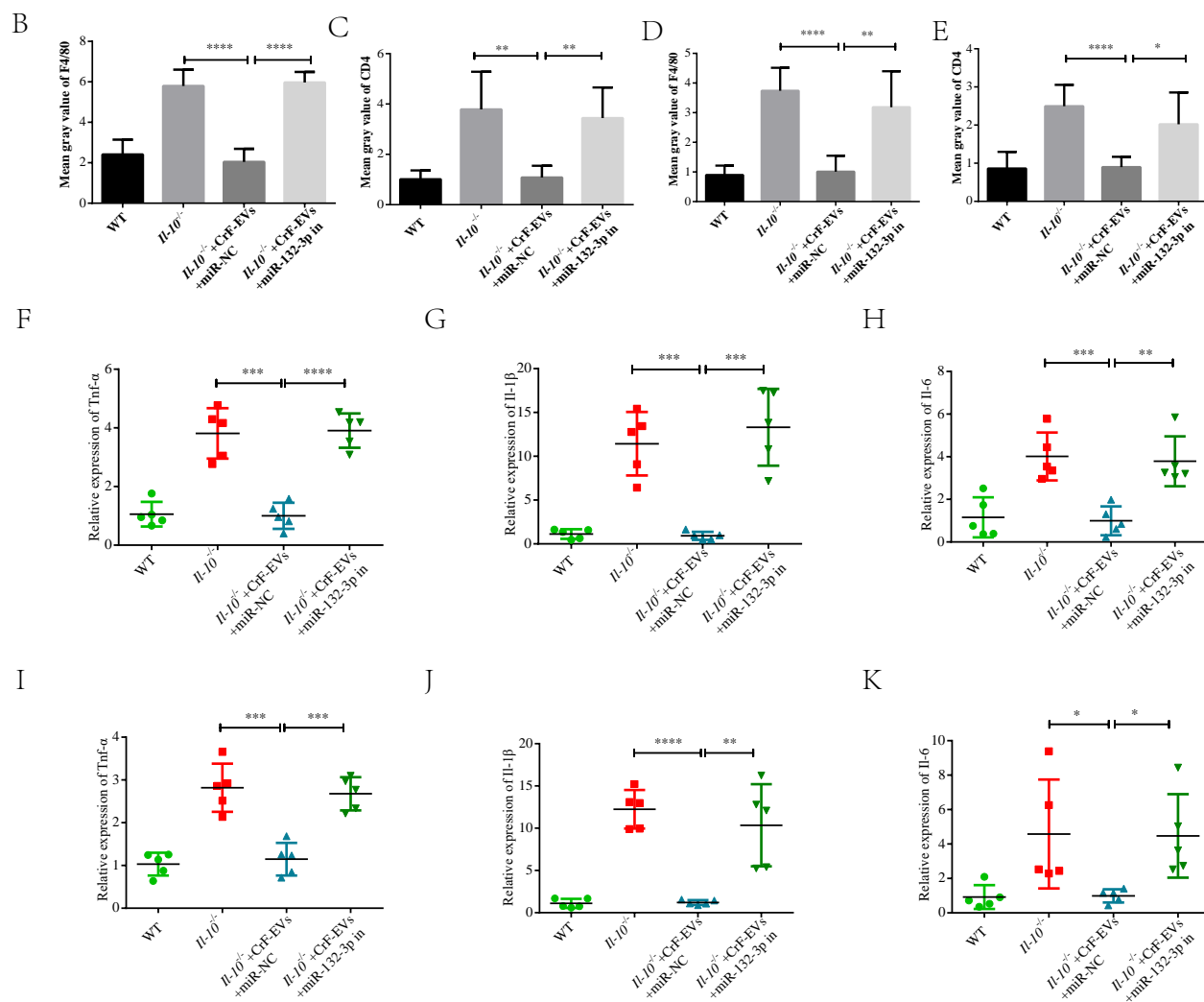

Supplement: Supplementary file 11 — Supporting Information [file CTM2-14-e70086-s002.pdf]

A

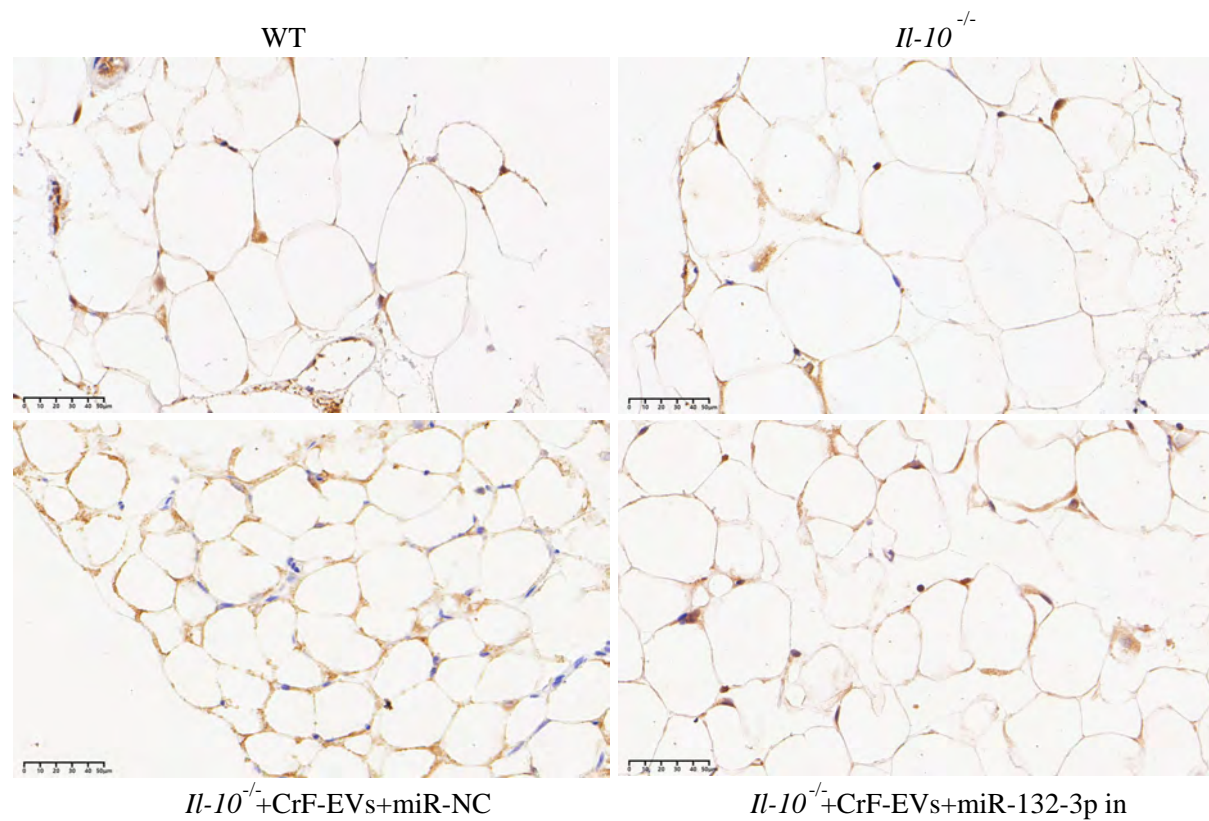

B

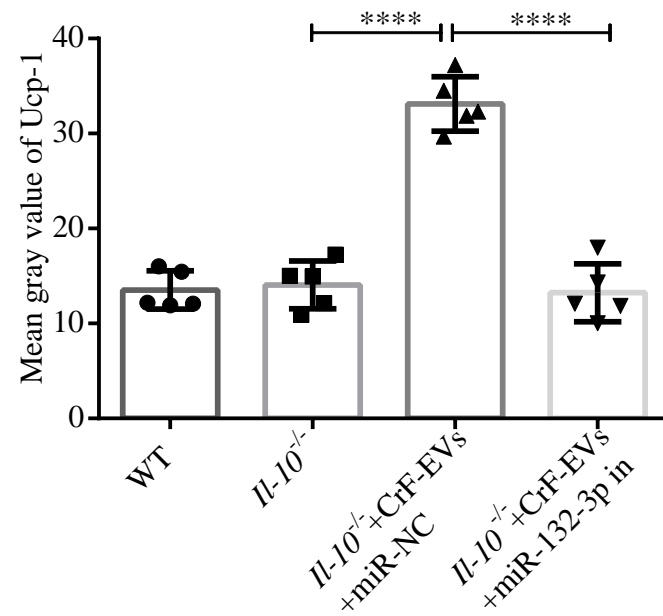

Supplement: Supplementary file 12 — Supporting Information [file CTM2-14-e70086-s004.pdf]

A

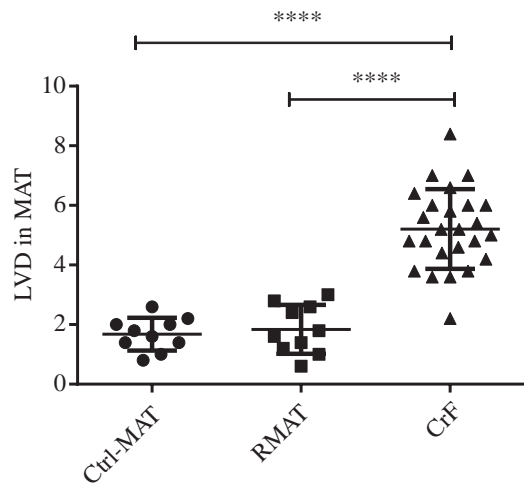

B

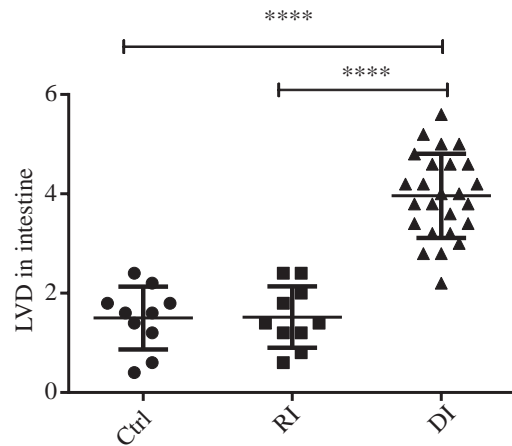

C

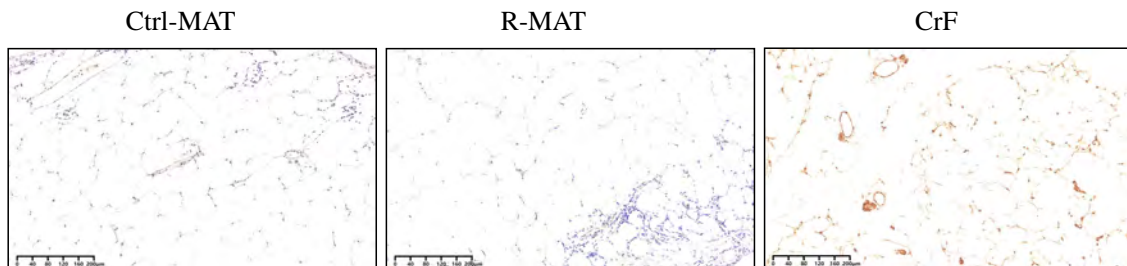

Supplement: Supplementary file 13 — Supporting Information [file CTM2-14-e70086-s007.pdf]
