# Supplementary material for: Extracellular vesicles derived from creeping fat stem cells promote lymphatic function and restrain inflammation of Crohn's disease
Source: Clin Transl Med. 2024 Dec 2;14(12):e70086. doi: 10.1002/ctm2.70086 (PMC11612264; doi:10.1002/ctm2.70086)
Supplement: Supplementary file 14 — Supporting Information [file CTM2-14-e70086-s006.docx]

**Supplemetary Materials**

**Adipose Stem Cell-derived extracellular vesicle miR-132-3p from Creeping fat Regulates Lymphatic Functions and Restrains Mesenteritis and Colitis in Crohn’s disease**

Weigang Shu,^1 #^ Yongheng Wang,^1 #^ Mengfan Chen,^1^ Xiaoli Zhu,^1^ Fangtao Wang,^2^ Chunqiu Chen,^2^ Peng Du,^3^ Alexandra Bartolomucci ^4^, Xin Su, ^4^*, Xiaolei Wang, ^1^*

1 Department of Gastroenterology, Shanghai Tenth People’s Hospital, School of Medicine, Tongji University, Shanghai 200072, China

2 Center for Difficult and Complicated Abdominal Surgery, Shanghai Tenth People’s Hospital, School of Medicine, Tongji University, Shanghai 200072, China

3 Department of Colorectal Surgery, Xinhua Hospital, Shanghai Jiaotong University, School of Medicine, Shanghai 200092, China

4 Cancer Research Program, Research Institute of McGill University Health Center, Montreal, Quebec, H4A 3J1 , Canada

^#^ The first two authors contributed equally to this work

*** Correspondence**

Xiaolei Wang. MD, PhD. Department of Gastroenterology, Shanghai Tenth People’s Hospital, School of Medicine, Tongji University, Shanghai 200072, China. E-mail: [wangxiaolei@tongji.edu. cn](mailto:wangxiaolei@tongji.edu.%20cn).

Xin Su. MD, PhD. Cancer Research Program, Research Institute of McGill University Health Center, Montreal, Quebec, Canada. RI‐MUHC, 1001 Decarie Blvd., Block ELab #E02.4134, Montreal, Quebec H4A 3J1, Canada. Email: xin.su3@mail.mcgill.ca

**MATERIALS AND METHODS**

Reagents

Human lymphatic endothelial cells (HLECs), endothelial cell medium (ECM) supplied with 1% endothelial cell growth factor (ECGF), Penicillin-Streptomycin (P/S) and 5% fetal bovine serum (FBS) were purchased from Wuhan Saios Company. The CCK-8 kit was purchased from the Shanghai Yeasun Biotechnology Company, Ltd. (Shanghai, China). Matrigel was purchased from BD Biosciences (Bedford, MA, USA). The inhibitor of ERK1/2 pathway (U0126) were purchased from Selleckchem (Shanghai, China). The miRNA mimics, inhibitor, scrambled miRNA control and transfection reagent were brought from Guangzhou Ribobio Co., Ltd. Plasmids with RASA1 overexpression were designed and synthesized by Genomeditech (Shanghai, China). Antibodies used in western blot: anti-RASA1 (#ab40677), anti-TSG101 (#ab125011), anti-CD9 (#ab236630), anti-CD63 (#ab134054), anti-ALIX (#ab275377), anti-Tubulin (#ab68193), anti-GRP78 (#ab21685) and anti-CALNEXIN (#ab22595) were brought from Abcam (Cambridge, MA, USA), anti-Phospho-p44/42 mitotic activated protein kinase (MAPK) (ERK1/2) (#9101) and anti-p44/42 MAPK (ERK1/2) (#9102) were brought from Cell Signaling Technology (CST, Danvers, MA, USA).

Isolation and culture of ADSCs

ADSCs were isolated according to our previously reported methods [1]. Briefly, the harvested adipose tissues were washed in phosphate-buffered saline [PBS] to remove erythrocytes and digested with 0.1% collagenase type I [Sigma-Aldrich] at 37°C under constant shaking. After centrifugation, the supernatant was removed, and the pelleted cells were resuspended and maintained in dulbecco's modified eagle medium (DMEM)/high-glucose medium containing 10% fetal bovine serum [FBS] and 1% penicillin/streptomycin at 37°C in a humidified 5% CO_2_ incubator. Most ADSCs were used passage 3 to passage 6.

Isolation and identification of EVs

EVs were isolated and purified as previously described [2]. Briefly, the control (Ctrl)-ADSCs and CrF-ADSCs were cultured in an EVs-free medium for 48 h. The culture supernatant was collected and centrifuged at 4 °C at 300 g for 10 min and then 3000 g for 30 min to remove cells and cell debris. The samples were then centrifuged at 10000 g for 30 min and 100000 g for 70 min to isolate the EVs. Finally, the pellets of EVs were centrifuged for another 70 min at 100000 g before being stored at –80°C.

The morphology of the isolated CrF-EVs and Ctrl-EVs were imaged by transmission electron microscopy [TEM; FEI Tecnai Spirit 120 kV]. The diameter of the EVs was detected using an LM10 NTA device [Malvern]. EVs were then dissolved in radio-immuno-precipitation assay [RIPA] buffer, and protein concentration was determined using a bicinchoninic acid [BCA] protein assay kit [Thermo Fisher Scientific]. Expression of EV-specific surface markers [TSG101, CD9, CD63 and ALIX] was determined using western blotting.

Culture of human lymphatic endothelial cells (HLECs)

ECM supplemented with 5% FBS, 1% P/S (100 U/mL), and 1% ECG was used to culture HLECs. After the HLECs reach 70% confluences, different conditions of EVs were added at the final concentration of 10 μg/mL for 24h. Then the conditioned medium and cells were collected respectively for further experiments.

EVs uptake by LECs and tracking *ex vivo*

PKH26 [Sigma Aldrich] or DiR dye [Invitrogen] was used to label EVs according to the manufacturer’s instructions. Briefly, 2mg/mL dye was added to the EVs solution and incubated for 10 min. The incubation solution was then centrifuged at 100000g for 70min to remove excess dye. To visualize EVs uptake by LECs, 10 µg/mL PKH26-labelled EVs were incubated with HLECs for 24 h. The cells were then washed with PBS to remove EVs that were not internalized by HLECs and stained with 4ʹ,6-diamidino-2-phenylindole [DAPI; Thermo Fisher Scientific]. Finally, a confocal imaging system [Zeiss LSM880] was used to observe EVs internalization by HLECs. To verify EVs distribution in the colon and mesentery, 200 µg DiR-labelled EVs were tail-vein injected into mice. Twelve hours later, the mice were sacrificed, and the fluorescence signal was detected using an IVIS Lumina II *in vivo* imaging system [PerkinElmer, Thermo Fisher].

EVs treatment in *Il-10^-/-^* mice

In our laboratory, *Il-10*^-/-^ mice displayed spontaneous colitis and mesenteritis with mesenteric hypertrophy at the 15^th^ week. To evaluate the effects of CrF-EVs in disease procession, EVs were injected into 15-week *Il-10*^-/-^ mice through tail-vein for 4 weeks. In brief, EVs were given in day 2 and day 5 of each week with 200 μg per mouse each time. Body weight was recorded every week during treatment. Mice were sacrificed at end of the 19^th^ week, and the colon and MAT samples were collected for further study. The animal study protocol was approved by the Ethics Committee of Shanghai Tenth People’s Hospital [2021JLHDWLSZ-0010].

Cell transfections and transductions

The negative control [miR-NC] and inhibitor of miR-132-3p [miR-132-3p in] were purchased from RiboBio and transfected into cells using Lipofectamine 2000 reagent [Invitrogen] according to the manufacturer’s instructions when cell confluence reached 50 %. The final concentration of the miR-132-3p inhibitor was 100 nM.

Cell Proliferation Assay

The CCK-8 kit was used to evaluate cell viability and the optical density value (OD value) which was in direct proportion to the number of living cells was used to determine cell viability. A microplate reader was used to measure the absorbance at 450 nm. All experiments were performed in triplicate.

Cell migration assay

LECs migration assay was performed using transwell chambers with inserts of 8 µm pore size (Corning Costar) as described previously [2]. 5×10^4^ LECs suspended in serum-free media were plated into the upper chamber for migration assay following treatment with indicated reagents, and media supplemented with 5 % FBS was placed into the lower chamber. After 12 hours, the cells that had migrated through the membrane to the lower surface were fixed, stained and counted.

Tube formation assay

Matrigel tube formation assays were carried out as previously described [2]. Briefly, matrigel was placed in 96-well plates (100 μL/well) and allowed to gel at 37°C for 30 minutes. 2×10^4^ LECs were then seeded onto the coated wells and cultured with 100μL of ECM, ECM containing indicated reagents at 37°C for 2h under 5% CO_2_, and then LEC tube formation was assessed by microscopy, and each well was photographed. The total tube length was calculated using the Image J software (National Institutes of Health, Bethesda, MD, USA).

Immunofluorescence (IF) staining

MAT and intestine samples were fixed in 4% paraformaldehyde, dehydrated, paraffin embedded, and sectioned at 5μm thickness. The following primary antibodies were used to detect the expression of specific proteins: rabbit anti-Lyve-1 (1:500, ab218535, Abcam), rabbit anti-Rasa1 (1:100, ab40677, Abcam). Cy3 (red)- or Alexa 488 (green)-labeled secondary antibodies (Servicebio) and DAPI (Servicebio, Wuhan, China) were used. IF was visualized with a fluorescence microscope (Nikon). The colocalization analysis was calculated by Manders’ colocalization coefficients based on pixel- intensity- correlation measurement.

Quantitative Real-Time Polymerase Chain Reaction (qRT-PCR)

The total RNA of MAT samples and HLECs were isolated with TRIzol reagent (Ambion, Thermo Fisher, Germany). The concentration and purity were measured with a NanoVue spectrophotometer (GE Healthcare, Germany). The cDNA was subsequently synthesized by PrimeScript™ RT reagent kit (Takara, Ostu, Shiga, Japan) according to the manufacturer’s instructions. qRT–PCR was carried out via a 7900HT Fast real-time PCR system (Applied Biosystems, Foster City, CA, USA) in conjunction with TB Green Premix Ex Taq™ II (Takara, Ostu, Shiga, Japan). The relative mRNA expression levels were evaluated by the 2^−ΔΔCT^ method and normalized to GAPDH expression. Sequences of PCR primers in this study are listed in the as below.

Sequences of PCR primers in this study

| **Gene** | **Forward primer (5'--3')** | **Reverse primer (5'--3')** |
| --- | --- | --- |
| Mouse-Gapdh | AGGTCGGTGTGAACGGATTTG | TGTAGACCATGTAGTTGAGGTCA |
| Mouse-Tnf-α | CATCTTCTCAAAATTCGAGTGACAA | TGGGAGTAGACAAGGTACAACCC |
| Mouse-Il-1β | GAGGACATGAGCACCTTCTTT | GCCTGTAGTGCAGTTGTCTAA |
| Mouse-Il-6 | TCTATACCACTTCACAAGTCGGA | GAATTGCCATTGCACAACTCTTT |
| Human-GAPDH | GGAGCGAGATCCCTCCAAAAT | GGCTGTTGTCATACTTCTCATGG |
| Human-ADIPONECTIN | AACATGCCCATTCGCTTTACC | TAGGCAAAGTAGTACAGCCCA |
| Human-VEGF-C | AGACTCAATGCATGCCACG | TTGAGTCATCTCCAGCATCC |
| Human-TNF-α | GAGGCCAAGCCCTGGTATG | CGGGCCGATTGATCTCAGC |
| Human-IL-6 | ACTCACCTCTTCAGAACGAATTG | CCATCTTTGGAAGGTTCAGGTTG |

Immunohistochemistry (IHC) Staining

For IHC staining, MAT and intestine samples were collected and fixed with 10% paraformaldehyde for 24h, embedded with paraffin and sliced into 5μm-thick sections. After rehydration with 80% methanol, PBS and PBS with 12% BSA, the sections were incubated with primary antibodies overnight at 4°C. The next day, the slides were washed with TBS-Tween and incubated with secondary antibodies. Standard immunohistochemical staining were performed as described previously. Primary antibody LYVE-1 was purchased from Abcam (Cambridge, UK) and used as 1:200. The lymphatic vessel density (LVD, per mm^2^) were represent by average value of six random fields with the highest density (“hot areas”) of LYVE-1^+^ vessels in the intestine and MAT by light microscopy at 200 × magnification.

Western Blotting Analysis

After quantification of the protein samples using a Micro BCA™ Protein Assay Kit (Beyotime, Shanghai, China), 10µg of proteins were subjet to electrophoresis on 7.5% or 10% SDS–PAGE gels, and transferred to PVDF membranes (Millipore, Billerica, MA, USA). After blocking, the membranes were incubated at 4°C overnight with the following primary antibodies: TSG101 (1:1000), CD9 (1:1000), CD63 (1:1000), ALIX (1:1000), Tubulin (1:5000), RASA1 (1:1000), ERK1/2 (1:1000), *p*-ERK1/2 (1:1000) and then incubated with the aforementioned anti-mouse or anti-rabbit secondary antibodies (1:5000) at 37°C for 1 h. Finally, the blots were scanned using an Odyssey two‐colour infrared laser imaging system (LI‐COR Biosciences, Lincoln, NB, USA). The intensity of the selected bands was quantified and analyzed using ImageJ software.

RNA extraction and library construction

Total RNA was extracted using mirVana miRNA Isolation Kit (Ambion) according to the manufacturer’s protocol. Quantitation of total RNA was carried out using the Nanodrop 2000 (Thermo Fisher Scientific Inc., USA). RNA integrity was assessed by Agilent 2100 Bioanalyzer (Agilent Technology, USA). 1 μg total RNA of each sample was used for the small RNA library construction using TruSeq Small RNA Sample Prep Kits (Cat. No. RS-200-0012, Illumina, USA.) following the manufacturer’s recommendations. Briefly, total RNA were ligated to adapters at each end. Then the adapter-ligated RNA were reverse transcribed to cDNA and performed PCR amplification. The PCR products ranging from 140–160 bp were isolated and purified as small RNA libraries. Library quality was assessed on the Agilent Bioanalyzer 2100 system using DNA High Sensitivity Chips. The libraries were finally sequenced using the Illumina HiSeq X Ten platform. 150 bp paired-end reads were generated. The small RNA sequencing and analysis were conducted by OE Biotech Co., Ltd. (Shanghai, China).

miRNA target prediction

The target genes of the miRNAs were predicted using the bioinformatics databases TargetScan, miRDB, miRanDa and mirTarBase. The common 12 differentially expressed genes from the four databases were further analyzed. Gene Ontology (GO) enrichment analyses were used to identify the significant pathways. *p* < 0.05 was set as the cutoff criterion for significant enrichment.

EV transfection of miRNA

The miRNA-132-3p inhibitor transfection into CrF-EVs was performed according to the Exo-Fect^TM^-miRNA transfection kit. Briefly, miR-132-3p inhibitor was incubated with Exo-Fect miRNA transfection reagent at room temperature (TM) for 15min, then the isolated CrF-EVs (200μg in a total volume of 100μL) was added and incubated at 37℃ for 1h. After that, this solution system above was transferred to the included pre-washed spin-column and incubated with gentle rotation at TM for 10min, then centrifugation at 1000g for 30sec was performed and the miR-132-3p inhibitor-loaded CrF-EVs were collected.

Endoscopic procedures

The experimental endoscopy setup denoted ‘‘Coloview system’’, consisted of a miniature endoscope (scope 1.9 mm outer diameter), a xenon light source, a triple chip camera, and an air pump (all from Karl Storz, Tuttlingen, Germany) to achieve regulated inflation of the mouse colon. The endoscopic procedure was viewed on a color monitor and digitally recorded on tape (DSR-20MDP; Sony, Cologne, Germany). The details of endoscopic scores were supplied as below.

Endoscopic colitis grading in this study

| Endoscopic colitis grading | | | | |
| --- | --- | --- | --- | --- |
| Colon Thickening | Transparent  (0) | Moderate  (1) | Marked  (2) | Intransparent  (3) |
| Vascular changes | Normal  (0) | Moderate  (1) | Marked  (2) | Bleeding  (3) |
| Fibrin visible | None  (0) | Little  (1) | Marked  (2) | Extreme  (3) |
| Mucosal granularity | None  (0) | Moderate  (1) | Marked  (2) | Extreme  (3) |
| Stool consistence | Normal  (0) | Still shaped  (1) | Unshaped  (2) | Spread  (3) |

Fluorescence lymphangiography

A long-chain fluorescein isothiocyanate(FITC)-labelled fatty acid [BODIPY FLC16; Thermo] was administered orally to wild-type (WT) and *Il-10*^-/-^ mice to investigate lymphatic drainage as previously reported [3], respectively. In total, 1 mg product was dissolved in 5 mL vegetable oil, and each mouse received 100 µL solution via oral administration. Fluorescence images of the mesenteric lymphatic drainage were obtained on a dissecting microscope 6 h after administration [Leica]. As previously defined, lymphatic drainage functionality was assessed by counting the number of functional lymphatic vessels per field in the mesentery.

Lymphatic drainage assay *in vivo* by Evans blue

Ten micrograms (1%) of Evans blue dye (Sigma-Aldrich, St. Louis, MO, USA) in 10μL of PBS was injected into the rectal mucosa of anaesthetized mice (N = 3 per group) using a Hamilton syringe according to previous studies. Mice were sacrificed after 16h. Evans blue dye was extracted from the distal colon tissues of comparable weight by incubating at 55℃ overnight in 1ml formamide (Sangon Biotech, Shanghai, China). The background-subtracted absorbance at 630nm was measured with a microplate reader (Biotek, Vermont, USA). The concentration of extracted dye was calculated using a standard curve and presented as the absolute amount of dye remaining in the colons.

**Fig. S1 Analysis of DIR-labeled fluorescence signal in mesentery and colon of *Il-10*^-/-^ mice.** DIR-labeled fluorescence signal detected in colon (A) and mesentery (B) of *Il-10*^-/-^ mice .n=3 mice in each group

**Fig. S2 Changes in mesentery of mice.** (A) Analysis of adipocyte size in mesentery of mice. (B,C) IHC staining and gray value analysis of Ucp-1 in mesentery of mice. Data are expressed as means±SD. *****p*<0.0001 and n=5 mice in each group.

**Fig. S3 Analysis of immune cell infiltration in mice.** F4/80^+^ macrophages (A) and CD4^+^ T cells (B) in colon of mice. F4/80^+^ macrophages (C) and CD4^+^ T cells (D) in mesentery of mice. F4/80^+^ macrophages (E) and CD4^+^ T cells (F) in lymph node of mice. Data are expressed as means±SD. **p*<0.05, ***p*<0.01, ****p*<0.001, *****p*<0.0001 and n=5 mice in each group.

**Fig. S4 Analysis of serum inflammatory cytokines in mice.** Elisa detected serum (A) Tnf-α and (B) Il-6. Data are expressed as means±SD. ****p*<0.001, *****p*<0.0001 and n=5 mice in each group.

**Fig. S5 Subcutaneous adipose tissue derived EVs (SAT-EVs) displayed no difference in lymphatic functions compared with Ctrl-EVs.** (A) HLECs proliferation with different EVs coincubation. (B) HLECs migration and tube formation with different EVs incubation. (C,D) Quantification of HLECs migration (C) and tube formation (D) with different EVs incubation. (E) ELISA analyzed CCL21 secretion by different EVs incubation. Data are expressed as means±SD.

**Fig. S6 miR-10b-5p. miR-873-3p and miR-365b-5p exerted no influences on lymphatic functions.** (A) qPCR detected miR-10b-5p. miR-873-3p and miR-365b-5p in CrF-EVs and Ctrl-EVs. (B) qPCR detected miR-10b-5p. miR-873-3p and miR-365b-5p expression in HLECs after transfection with miRNA mimics. (C) HLECs migration and tube formation with different miRNA mimics transfection. (D,E) Quantification of HLECs migration (E) and tube formation (D) with different miRNA mimics transfection. (F) HLECs proliferation with with different miRNA mimics transfection. Data are expressed as means±SD. ***p*<0.01, ****p*<0.001.

**Fig. S7 qPCR detected miR-132-3p expression.** qPCR detected miR-132-3p expression in (A) CrF-ADSCs and (B) HLECs after miR-132-3p mimics transfection. Data are expressed as means±SD. ***p*<0.01, ****p*<0.001.

**Fig. S8 Western blot detected ERK1/2 phosphorylation with RASA1 overexpression plasmid transfection and ERK1/2 phosphorylation inhibitor U0126 pre-treatment.** (A-C) analysis of ERK1/2 phosphorylation after RASA1 overexpression. (D-F) analysis of ERK1/2 phosphorylation with U0126 pre-treatment. Data are expressed as means±SD. **p*<0.05, ***p*<0.01, ****p*<0.001, *****p*<0.0001 and n=5 mice in each group.

**Fig. S9** **CrF-EVs-miR-132-3p targeted Rasa1 expression of HLECs and promoted lymphangiogenesis *in vivo*.** (A) IF double staining of Lyve-1 and Rasa1 in the colon of *Il-10*^-/-^ mice. (B) IHC staining of Lyve-1 in the colon of *Il-10*^-/-^ mice. (C) LVD analysis in the colon of *Il-10*^-/-^ mice. Data are expressed as means±SD. *****p*<0.0001.

**Fig. S10 Analysis of adipocyte size in mesentery of mice.**

**Fig. S11 Analysis of immune cell infiltration in mice**. (A) F4/80 positive macrophages and CD4 positive T cells in the colon and mesentery. Analysis of F4/80^+^ macrophages (B) and CD4^+^ T cells (C) in colon of mice. F4/80^+^ macrophages (D) and CD4^+^ T cells (E) in mesentery of mice.(F-H) Relative mRNA expression of Tnf-α (F), Il-1β (G) and Il-6 (H) expression in colon. (I-K) Relative mRNA expression of Tnf-α (I), Il-1β (J) and Il-6 (K) expression in mesentery. Data are expressed as means±SD. **p*<0.05, ***p*<0.01, ****p*<0.001, *****p*<0.0001

**Fig. S12 IHC staining and gray value analysis of Ucp-1 in mesentery of mice.**

**Fig. S13 Analysis of LVD and IHC staining of CCL21 in mesentery samples.** (A,B) Analysis of LVD in (A) MAT and (B) intestine. (C) IHC staining of CCL21 in mesentery samples. Data are expressed as means±SD. *****p*<0.0001.

**Table S1** **Clinical characteristics of patients for MAT collection in this study (Cohort 1)**

|  | Control (N=10) | Patients with CD  (N=25) |
| --- | --- | --- |
| Gender (male/female) | 6/4 | 16/9 |
| Age (years) | 42.50 ± 7.36 | 40.40 ± 13.08 |
| BMI (kg/m^2^) | 20.10 ± 2.26 | 19.68 ± 3.22 |
| Smoking history, n (%) | 4 (40.00) | 11 (44.00) |
| Disease duration (years) | - | 28.24 ± 7.13 |
| Age at diagnosis, n (%) |  |  |
| ≤ 16 years |  | 0 (00.00) |
| 17 - 40 years |  | 18 (72.00) |
| > 40 years |  | 7 (28.00) |
| Location, n (%) |  |  |
| ileal |  | 10 (40.00) |
| colonic |  | 7 (28.00) |
| ileocolonic |  | 8 (32.00) |
| Behavior, n (%) |  |  |
| non-stenotic, non-fistulizing |  | 5 (20.00) |
| stenotic |  | 14 (56.00) |
| fistulizing |  | 6 (24.00) |
| Previous CD-related surgery, n (%) |  | 7 (28.00) |
| Indications for surgery  (Stenosis/Abscess/Inflammation), n |  | (14/9/2) |
| Medications, n(%) |  |  |
| 5-ASA |  | 15 (48.00) |
| Antibiotics |  | 7 (16.00) |
| Steroids |  | 6 (24.00)- |
| Immunosuppression |  | 4 (16.00) |
| Biological therapy |  | 0 (00.00) |
| No treatment |  | 3 (12.00) |

Abbreviations: BMI, body mass index; CD, Crohn’s disease

^a^*p* < 0.05 significant differences between Control and patients with CD.

**Table S2** **Clinical characteristics of patients for serum analysis in this study (Cohort 2)**

|  | Control (N=10) | Patients with CD  (N=30) |
| --- | --- | --- |
| Gender (male/female) | 6/4 | 16/14 |
| Age (years) | 38.40± 9.26 | 40.30 ± 13.08 |
| BMI (kg/m^2^) | 20.60 ± 2.71 | 19.87 ± 3.24 |
| Smoking history, n (%) | 5 (50.00) | 19 (54.29) |
| Disease duration (years) | - | 24.31 ± 8.23 |
| Age at diagnosis, n (%) |  |  |
| ≤ 16 years |  | 0 (00.00) |
| 17 - 40 years |  | 21 (70.00) |
| > 40 years |  | 9 (30.00) |
| Location, n (%) |  |  |
| ileal |  | 15 (50.00) |
| colonic |  | 9 (30.00) |
| ileocolonic |  | 6 (20.00) |
| Behavior, n (%) |  |  |
| non-stenotic, non-fistulizing |  | 12 (40.00) |
| stenotic |  | 15 (50.00) |
| fistulizing |  | 3 (10.00) |
| Disease severity, n(%) |  |  |
| Remission |  | 20 (66.67) |
| Active |  | 10 (33.33) |
| Medications, n(%) |  |  |
| 5-ASA |  | 15 (50.00) |
| Antibiotics |  | 6 (20.00) |
| Steroids |  | 4 (13.33)- |
| Immunosuppression |  | 4 (13.33) |
| Biological therapy |  | 0 (00.00) |
| No treatment |  | 3 (10.00) |
| C-reactive protein (mg/dL) | 4.83 ± 1.50 | 39.21 ± 17.05^a^ |
| Serum levels IL-6 (pg/mL) | 2.37 ± 1.34 | 28.72 ± 12.02^a^ |

Abbreviations: BMI, body mass index; CD, Crohn’s disease

^a^*p* < 0.05 significant differences between Control and patients with CD..

**References**

1. Zhang L, Ye C, Li P, et al. ADSCs stimulated by VEGF-C alleviate intestinal inflammation via dual mechanisms of enhancing lymphatic drainage by a VEGF-C/VEGFR-3-dependent mechanism and inhibiting the NF-κB pathway by the secretome. *Stem Cell Res Ther* 2022;13(1):448.
2. Wang X, Wang H, Cao J, Ye C. [Exosomes from Adipose-Derived Stem Cells Promotes VEGF-C-Dependent Lymphangiogenesis by Regulating miRNA-132/TGF-β Pathway.](https://pubmed.ncbi.nlm.nih.gov/30134228/) *Cell Physiol Biochem* 2018;49(1):160-171.
3. Yin Y, Yang J, Pan Y, et al.. [Chylomicrons-Simulating Sustained Drug Release in Mesenteric Lymphatics for the Treatment of Crohn's-Like Colitis.](https://www.geenmedical.com/article?id=32978613&type=true) *J Crohns Colitis* 2021;15(4):631-646.
